# Identification of a novel α(1→6) mannopyranosyltransferase MptB from *Corynebacterium glutamicum* by deletion of a conserved gene, *NCgl1505*, affords a lipomannan- and lipoarabinomannan-deficient mutant

**DOI:** 10.1111/j.1365-2958.2008.06265.x

**Published:** 2008-06

**Authors:** Arun K Mishra, Luke J Alderwick, Doris Rittmann, Cindy Wang, Apoorva Bhatt, William R Jacobs, Kuni Takayama, Lothar Eggeling, Gurdyal S Besra

**Affiliations:** 1 School of Biosciences, University of Birmingham, EdgbastonBirmingham B15 2TT, UK; 2 Institute for Biotechnology 1, Research Centre JuelichD-52425 Juelich, Germany; 3 Mycobacteriology Research Laboratory, William S. Middleton Memorial Veterans HospitalMadison, WI 53705, USA; 4 Howard Hughes Medical Institute, Department of Microbiology and Immunology, Albert Einstein College of MedicineBronx, NY 10461, USA

## Abstract

*Mycobacterium tuberculosis* and *Corynebacterium glutamicum* share a similar cell wall structure and orthologous enzymes involved in cell wall assembly. Herein, we have studied *C. glutamicum* NCgl1505, the orthologue of putative glycosyltransferases Rv1459c from *M. tuberculosis* and MSMEG3120 from *Mycobacterium smegmatis.* Deletion of *NCgl1505* resulted in the absence of lipomannan (Cg-LM-A), lipoarabinomannan (Cg-LAM) and a multi-mannosylated polymer (Cg-LM-B) based on a 1,2-di-*O*-C_16_/C_18:1_-(α-D-glucopyranosyluronic acid)-(1→3)-glycerol (GlcAGroAc_2_) anchor, while syntheses of triacylated-phosphatidyl-*myo*-inositol dimannoside (Ac_1_PIM_2_) and Man_1_GlcAGroAc_2_ were still abundant in whole cells. Cell-free incubation of *C. glutamicum* membranes with GDP-[^14^C]Man established that *C. glutamicum* synthesized a novel α(1→6)-linked linear form of Cg-LM-A and Cg-LM-B from Ac_1_PIM_2_ and Man_1_GlcAGroAc_2_ respectively. Furthermore, deletion of *NCgl1505* also led to the absence of *in vitro* synthesized linear Cg-LM-A and Cg-LM-B, demonstrating that NCgl1505 was involved in core α(1→6) mannan biosynthesis of Cg-LM-A and Cg-LM-B, extending Ac_1_PI[^14^C]M_2_ and [^14^C]Man_1_GlcAGroAc_2_ primers respectively. Use of the acceptor α-D-Man*p*-(1→6)-α-D-Man*p-O*-C_8_ in an *in vitro* cell-free assay confirmed NCgl1505 as an α(1→6) mannopyranosyltransferase, now termed MptB. While Rv1459c and MSMEG3120 demonstrated similar *in vitro*α(1→6) mannopyranosyltransferase activity, deletion of the *Rv1459c* homologue in *M. smegmatis* did not result in loss of mycobacterial LM/LAM, indicating a functional redundancy for this enzyme in mycobacteria.

## Introduction

The taxon *Corynebacterineae* belongs to the Actinomycetes family which includes human pathogens, such as *Mycobacterium tuberculosis*, *Mycobacterium leprae* and *Corynebacterium diphtheriae*, the causal agents of tuberculosis, leprosy and diphtheria respectively (Coyle and Lipsky, 1990; Bloom and Murray, 1992). Some animal pathogens, for instance, *Corynebacterium pseudotuberculosis* and *Corynebacterium matruchotii* (Coyle and Lipsky, 1990; Funke *et al*., 1997; Stackebrandt *et al*., 1997), also belong to the *Corynebacterianeae*. In addition, the family member *Corynebacterium glutamicum* is widely used for the industrial production of amino acids (Eggeling and Bott, 2005). These bacilli share a unique cell wall ultra-structure that is composed of a mycolyl-arabinogalactan-peptidoglycan (mAGP) complex (Daffé*et al*., 1990; McNeil *et al*., 1990; 1991; Besra *et al*., 1995; Brennan, 2003; Dover *et al*., 2004). The esterified mycolates of the mAGP complex are considered to be packed side by side and are intercalated by lipids and glycolipids. This combined lipid structure gives rise to an asymmetric bilayer critical for the survival of these organisms (Minnikin *et al*., 2002).

In addition to the mAGP complex, other glycolipids, such as phosphatidyl-*myo*-inositol (PI) mannosides (PIMs) and lipoglycans, termed lipomannan (LM) and lipoarabinomannan (LAM), are also found in this outer leaflet (Hill and Ballou, 1966; Brennan and Ballou, 1967; 1968; Brennan and Nikaido, 1995; Besra *et al*., 1997; Morita *et al*., 2004). However, LM and LAM possess important physiological functions, and play key roles in the modulation of the host response during infection (Schlesinger *et al*., 1994; Chatterjee and Khoo, 1998; Nigou *et al*., 2002; Maeda *et al*., 2003). The modulation of the immune response by LAM has been attributed to its terminal-capping motif (Nigou *et al*., 2002; 2003). Different permutations of LAM capping have been found in *Mycobacterium* strains, including ManLAM (Chatterjee *et al*., 1993; Khoo *et al*., 1995), PILAM (Gilleron *et al*., 1997) and (non-capped) LAM (Guerardel *et al*., 2002). Slow-growing mycobacteria, such as *M. tuberculosis* and *M. leprae*, produce ManLAM, which enables them to infect macrophages and dendritic cells (Schlesinger *et al*., 1994; Tascon *et al*., 2000). ManLAM inhibits the production of proinflammatory cytokines, such as IL-12 and TNF-α and inhibits phagosomal maturation (Knutson *et al*., 1998; Nigou *et al*., 2002; Fratti *et al*., 2003), while PILAM from the non-pathogenic fast-growing *Mycobacterium smegmatis* strain induces the proliferation of these cytokines (Adams *et al*., 1993; Gilleron *et al*., 1997).

**Fig. 1:**
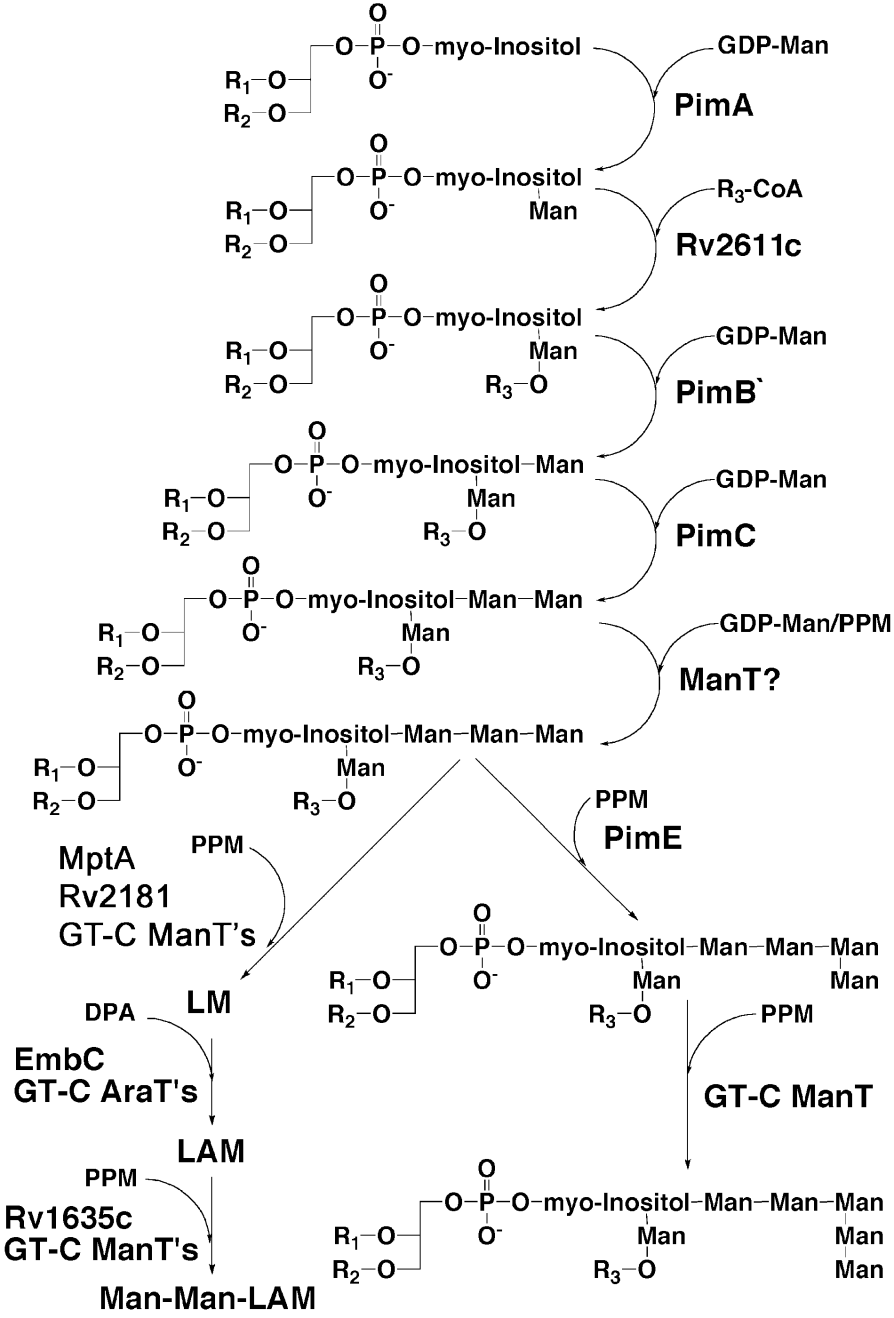
Schematic representation of the current understanding of the LM and LAM biosynthetic pathway in *M. tuberculosis*. ManT, mannosyltransferase; AraT, arabinosyltransferase; PPM, polyprenyl-1-monophosphorylmannose; DPA, decaprenyl-1-monophosphoryl-arabinose; R1, R2 and R3 represent acyl groups.

The current model of lipoglycan biosynthesis follows a linear pathway, PI→PIM→LM→LAM (Besra and Brennan, 1997) (Fig. 1). PI is glycosylated by an α-mannopyranosyl (Man*p*) residue catalysed by PimA (Rv2610c), which transfers Man*p* from GDP-mannose to the 2-position of PI to form PIM1 (Kordulakova *et al*., 2002). PIM1 is further glycosylated by PimB (Rv0557), which may occur before, or after acylation of PIM_1_ by Rv2611c (Kordulakova *et al*., 2003) and results in the formation of Ac_1_PIM_2_ (Schaeffer *et al*., 1999). However, recently, this second mannosylation step in the biosynthesis of Ac_1_PIM_2_ has now been shown to be catalysed by PimB′ (Rv2188c, NCgl2106), while PimB (Rv0557, NCgl0452), now termed MgtA, is involved in synthesizing a novel mannosylated glycolipid, 1,2-di-*O*-C_16_/C_18:1_-(α-D-mannopyranosyl)-(1→4)-(α-D-glucopyranosyluronic acid)-(1→3)-glycerol (Man_1_GlcAGroAc_2_) (Tatituri *et al*., 2007; Lea-Smith *et al*., 2008; Mishra *et al*., 2008). The analysis of deletion mutants of *NCgl0452* and *NCgl2106* established that this glycolipid is further modified to produce a multi-mannosylated derivative, Man_12−20_GlcAGroAc_2_ (Cg-LM-B) which is coincident on SDS-PAGE with PI-based Cg-LM, which is now termed Cg-LM-A (Tatituri *et al*., 2007; Lea-Smith *et al*., 2008; Mishra *et al*., 2008). Previous studies have shown that RvD2-ORF1 from *M. tuberculosis* CDC1551, designated as PimC, catalysed further α-mannosylation of Ac_1_PIM_2_ resulting in Ac_1_PIM_3_ (Kremer *et al*., 2002). Recently, PimE (Rv1159) has been shown to be involved in higher PIM biosynthesis and directly in the biosynthesis of Ac_1_PIM_5_ (Morita *et al*., 2006); however, the enzyme responsible for the synthesis of Ac_1_PIM_4_ from Ac_1_PIM_3_ remains to be identified.

The point at which lipoglycan biosynthesis continues probably occurs after Ac_1_PIM_2_ and Man_1_GlcAGroAc_2_ in *C. glutamicum* (Gibson *et al*., 2003; Tatituri *et al*., 2007; Mishra *et al*., 2008), where a transition occurs from glycosyltransferases which utilize nucleotide sugars (i.e. GDP-Man) as substrate to glycosyltransferases which utilize polyprenyl-phosphate sugars (i.e. polyprenyl-phosphomannose, PPM) and which belong to the GT-C superfamily, and are membrane-bound (Liu and Mushegian, 2003). Recently, we (Mishra *et al*., 2007) and others (Kaur *et al*., 2007) reported a novel α-mannosyltransferase, MptA (Rv2174), involved in the latter stages of Ms-LM/LAM, Cg-LAM, Cg-LM-A and Cg-LM-B biosynthesis in *Corynebacterineae*. The core mannan backbone is further glycosylated by Rv2181 and results in the synthesis of α(1→2)-Man*p*-linked branches, characteristic of the mannan backbone in LM and LAM (Kaur *et al*., 2006). The mature LM is then elaborated with arabinose by the essential arabinofuranosyltransferase EmbC (G.S. Besra, unpubl. res.) to form LAM (Berg *et al*., 2005). Recently, a novel mannosyltransferase, Rv1635c (and MT1671), has been shown to add terminal Man*p* residues to the mature LAM in *M. tuberculosis* to form ManLAM (Dinadayala *et al*., 2006; Appelmelk *et al*., 2007). However, the enzyme involved in the early stages of linear LM/LAM mannan core biosynthesis through an α(1→6) mannosyltransferase prior to MptA remains to be identified (Fig. 1).

In this study, we have examined the function of *C. glutamicum NCgl1505*, and its orthologous genes *Rv1459c* of *M. tuberculosis* and *MSMEG3120* of *M. smegmatis* encoding a putative GT-C glycosyltransferase. The *NCgl1505* gene and its orthologues based on the results described below have been designated as *mptB*, as an acronym for mannopyranosyltransferase B. Null mutants of *C. glutamicum* together with *in vitro* cell-free assays established that NCgl1505 is a key α(1→6) mannosyltransferase involved in the initiation of core mannan biosynthesis of Cg-LM-A and Cg-LM-B from *Corynebacterineae* extending Ac_1_PIM_2_ and Man_1_GlcAGroAc_2_ respectively. In addition, the *M. tuberculosis* orthologue Rv1459c and *M. smegmatis* MSMEG3120 demonstrated α(1→6) mannosyltransferase activity in a membrane-based *in vitro* assay when utilizing a *C. glutamicum*Δ*mptB*Δ*mptA* double mutant complemented with either plasmid-encoded Rv1459c or MSMEG3120. Finally, using a *M. smegmatis* null mutant of MSMEG3120, we also demonstrate that the mycobacterial orthologue of NCgl1505 is functionally redundant.

## Results

### Genome locus and structural features of Rv1459c/NCgl1505

**Fig. 2:**
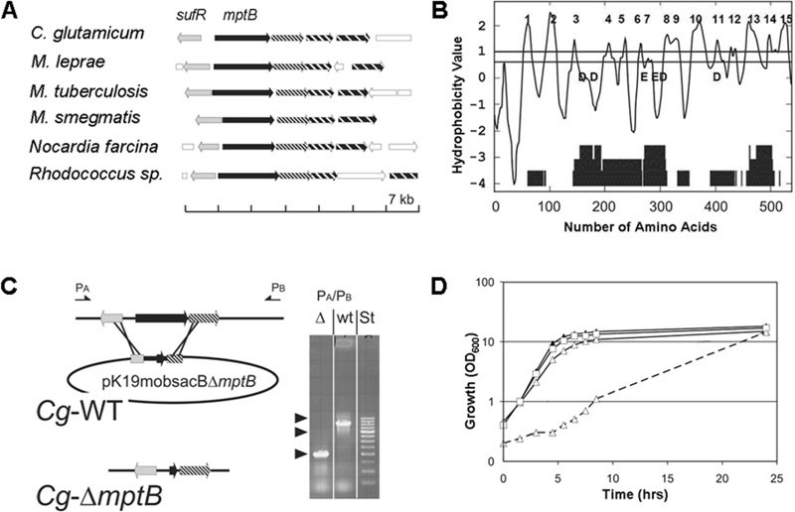
Generation of an in-frame deletion mutant of *C. glutamicum mptB*. A. The locus in the bacteria analysed consists of *mptB* which has in *C. glutamicum* the locus tag NCgl1505 and in *M. tuberculosis* Rv1459c. *sufR* encodes a transcriptional regulator in front of an operon of the SUF machinery of [Fe-S] cluster synthesis (Huet *et al*., 2006). The genomic region displayed encompasses 7 kb, and orthologous genes are highlighted accordingly. *Nocardia farcina*, *Nocardia farcina* IFM 10152; *Rhodococcus*, *Rhodococcus* sp. strain RHA1. B. MptB is a hydrophobic protein predicted to span the membrane 15 times and the transmembrane helices are numbered accordingly. The lower part of the figure shows the degree of conservation of the orthologues given in A as analysed by the dialign method (Morgenstern, 2004). Also shown is the approximate position of the fully conserved aspartyl (D) and glutamyl (E) residues. C. Strategy to delete Cg-*mptB* using the deletion vector pK19mobsacBΔ*mptB*. This vector carries 18 nucleotides of the 5′ end of Cg-*mptB* and 36 nucleotides of its 3′ end, thereby enabling the in-frame deletion of almost the entire Cg-*mptB* gene. The arrows marked PA and PB locate the primers used for the PCR analysis to confirm the absence of Cg-*mptB*. Distances are not drawn to scale. The results of the PCR analysis with the primer pair PA/PB are shown on the right. Amplification products obtained from the wild type (wt) were applied in the middle lane and that of the deletion mutant (Δ) in the left lane. ‘st’ marks the standard, where the arrowheads are located at 1.5, 1 and 0.5 kb. D. Growth of *C. glutamicum* on rich BHIS (solid lines). Wild-type *C. glutamicm*, filled triangle; *C. glutamicum*Δ*mptB*, open triangle; *C. glutamicum*Δ*mptB* pVWEx-Cg-*mptB*, open square. Growth of *C. glutamicum*Δ*mptB* on rich BHI medium are the open triangles with the broken line.

Glycosyltransferases belonging to the GT-C superfamily have been shown by us (Alderwick *et al*., 2005; Alderwick *et al*., 2006b; Mishra *et al*., 2007; Seidel *et al*., 2007) and others (Dinadayala *et al*., 2006; Kaur *et al*., 2006; 2007; Morita *et al*., 2006) to play important roles in the biosynthesis of the cell wall heteropolysaccharides arabinogalactan (AG), LM-A, LM-B and LAM in *Corynebacterinaeae*. Our attention was recently drawn to a putative glycosyltransferase encoded by *M. tuberculosis Rv1459c* and *C. glutamicum NCgl1505*, which are members of the GT-C family of glycosyltransferases. Orthologues of these genes are present in all *Mycobacterium* and *Corynebacterium* species as well as the sequenced *Nocardia farcinica* IFM 10152 and *Rhodococcus* sp. RHA1 strains (Fig. 2A). In addition, this gene is retained in *M. leprae*, supporting the hypothesis that NCgl1505 encodes for a protein possessing a vital function inherent to this group of bacteria.

The glycosyltransferase encoded by NCgl1505 is a polytopic membrane protein, which is comprised of 558 amino acid (aa) residues, and is predicted to encode 15 hydrophobic segments (HSs) (Fig. 2B). Rv1459c constitutes 591 aa, with the additional length mostly due to an extended loop between HSs 7 and 8. This loop extension is not present in *Mycobacterium paratuberculosis* or *M. smegmatis*. It contains a number of repeated Pro and Arg residues, and similarly highly charged repeat sequences are found in loop regions of other transporters, without having a specific function (Eng *et al*., 1998; Vrljic *et al*., 1999). The sequence identity of the orthologues NCgl1505 and Rv1459c is 37% (52% similarity) and can therefore be considered very high. The strongest conserved regions are found in loops connecting HSs and adjacent regions with intermediate hydrophobicity, like those between HSs 3–4, HSs 7–8 and HSs 13–14 (Fig. 2B). Within the highest conserved regions; five of the six fully conserved acidic Asp and Glu residues are located, given as D and E in Fig. 2B, which are known to play important roles as general bases and nucleophiles in enzyme catalysis. They are also retained in the MptB orthologue in *N. farcinica* IFM 10152 and *Rhodococcus* sp. RHA1 are therefore likely to be involved in catalysis, or in interactions with the sugar donor or acceptor (Liu and Mushegian, 2003). Interestingly, among the glycosyltransferases of *M. tuberculosis* and *C. glutamicum* previously identified (Alderwick *et al*., 2006a; Dinadayala *et al*., 2006; Kaur *et al*., 2006; Morita *et al*., 2006; Seidel *et al*., 2007), NCgl1505 and Rv1459c possess the highest identities to the recently identified mannosyltransferase MptA (Kaur *et al*., 2007; Mishra *et al*., 2007) and, based on the results described below, the *NCgl1505* gene and its orthologues have been designated as MptB.

### Construction and growth of *C. glutamicumΔmptB* and complemented strains

In order to delete *mptB* in *C. glutamicum*, the non-replicative plasmid pK19mobsacBΔ*mptB* was constructed carrying sequences adjacent to Cg-*mptB*. Using this vector, *C. glutamicum* was transformed to kanamycin resistance, indicating integration of the vector into the genome by homologous recombination (Fig. 2C). The *sacB* gene enables for selection of loss of vector in a second homologous recombination event, which can result either in the original wild-type genomic organization or in clones deleted of Cg-*mptB*. Ninety clones exhibiting the desired phenotype of vector loss (kanamycin-sensitive, sucrose-resistant) were analysed by PCR, but only one single colony was found to have Cg-*mptB* excised, whereas the others resulted in a wild-type genotype. The low number of recombinant knockouts indicates that the loss of Cg-*mptB* is apparently a disadvantage for cell viability, similar to that of previously observed mutants with altered mycolate (Gande *et al*., 2004) or arabinogalactan biosynthesis (Alderwick *et al*., 2006b). The resulting clone was subsequently termed *C. glutamicum*Δ*mptB* and confirmed by PCR with different primer pairs to have Cg-*mptB* deleted, whereas controls with *C. glutamicum* wild type resulted in the expected larger amplification product (Fig. 2C).

In liquid culture, growth of *C. glutamicum*Δ*mptB* was very poor. Only when rich brain heart infusion (BHI) medium was used was a growth rate of 0.13 h^−1^ obtained (Fig. 2D) in comparison with wild-type *C. glutamicum* growth rate of 0.31 h^−1^ (Mishra *et al*., 2007) and, on the same medium supplemented with 500 mM sorbitol (BHIS), the growth rate was 0.51 h^−1^, which is still lower than that of the wild type on this medium (0.70 h^−1^). *C. glutamicum*Δ*mptB* was transformed with pVWEx-Cg-*mptB* and the resultant complemented strain exhibited a growth rate of 0.66 h^−1^, almost superimposable to that of the wild type in BHIS medium.

### Polar lipid analysis of *C. glutamicum* and *C. glutamicumΔmptB*

Lyophilized cells were extracted using petroleum-ether and methanolic saline to initially recover apolar lipids. Further processing of the methanolic extract afforded the polar lipid fraction which was examined by two-dimensional thin-layer chromatography (2D-TLC). In both the wild-type *C. glutamicum* and *C. glutamicum*Δ*mptB*, Ac_1_PIM_2_ and Man_1_GlcAGroAc_2_ (Tatituri *et al*., 2007) were visualized either by α-naphthol/sulphuric acid (specific for sugars), 5% ethanolic molybdophosphoric acid (general lipid stain) (Fig. S1) or Dittmer and Lester reagent (specific for phospholipids). In both *C. glutamicum* and *C. glutamicum*Δ*mptB*, no products could be observed which correspond to higher PIMs (i.e. Ac_1_PIM_3_ through to Ac_1_PIM_6_) or higher mannose variants of Man_1_GlcAGroAc_2_ (Tatituri *et al*., 2007; Mishra *et al*., 2008). The presence of only Ac_1_PIM_2_ and Man_1_GlcAGroAc_2_, and the inability to synthesize Cg-LAM, Cg-LM-A and Cg-LM-B by *C. glutamicum*Δ*mptB* (as shown below) demonstrated that MptB is involved in the early steps of α(1→6) mannan core biosynthesis by extending the substrates Ac_1_PIM_2_ and Man_1_GlcAGroAc_2_.

### Analysis of lipoglycans from *C. glutamicum*, *C. glutamicumΔmptB* and *C. glutamicumΔmptB* pVWEx-Cg-*mptB*

**Fig. 3:**
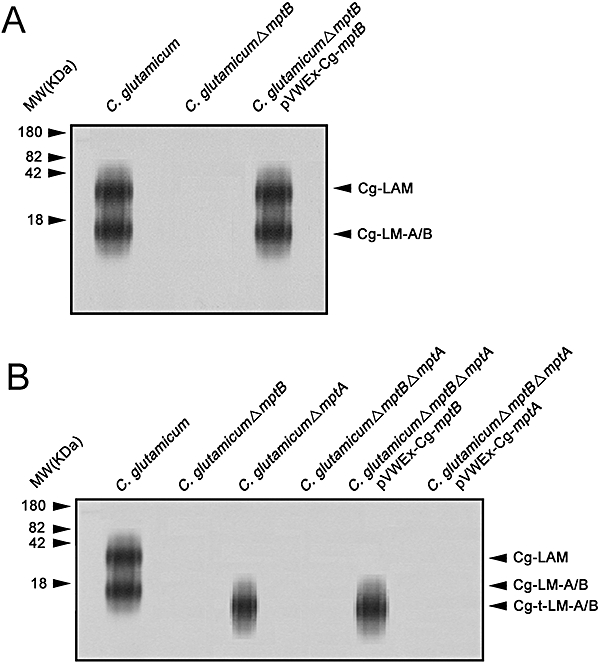
Lipoglycan profile of *C. glutamicum* strains analysed using SDS-PAGE and visualized using a Pro-Q emerald glycoprotein stain (Invitrogen) specific for carbohydrates. A. Lipolglycans extracted from *C. glutamicum*, *C. glutamicum*Δ*mptB* and *C. glutamicum*Δ*mptB* pVWEx-Cg-*mptB*. The major bands represented by Cg-LAM, Cg-LM-A and Cg-LM-B are indicated. B. *C. glutamicum*, *C. glutamicum*Δ*mptB*, *C. glutamicum*Δ*mptA*, *C. glutamicum*Δ*mptB*Δ*mptA*, *C. glutamicum*Δ*mptB*Δ*mptA* pVWEx-Cg-*mptB* and *C. glutamicum*Δ*mptB*Δ*mptA* pVWEx-Cg-*mptA*. The truncated version of Cg-LM-A/B is indicated as Cg-t-LM-A/B (Mishra *et al*., 2007). The four major bands represent glycoproteins of 180, 82, 42 and 18 kDa respectively.

Lipoglycans were extracted by refluxing delipidated cells in ethanol, followed by hot-phenol extraction, protease digestion and dialysis to remove impurities. The extracted lipoglycans were examined initially on 15% SDS-PAGE (Fig. 3A). Extracts from wild-type *C. glutamicum* showed the presence of Cg-LAM, Cg-LM-A and Cg-LM-B with the latter product based on previous results comigrating with Cg-LM-A (Tatituri *et al*., 2007; Mishra *et al*., 2008), while all of these lipoglycans were absent from *C. glutamicum*Δ*mptB*. Complementation of *C. glutamicum*Δ*mptB* by transformation with plasmid pVWEx-Cg-*mptB* restored the wild-type phenotype (Fig. 3A). In addition, transformation of *C. glutamicum*Δ*mptB* with plasmid pVWEx-Cg-*mptA* failed to restore the wild-type phenotype (data not shown).

### Construction and growth of *C. glutamicumΔmptAΔmptB* and complemented strains

As a result of the similarity of MptB with MptA, we wanted to exclude any possible interferences and constructed a strain of *C. glutamicum* deficient in *mptB* and *mptA*. For this purpose, *C. glutamicum*Δ*mptB* was transformed with plasmid pK19mobsacBΔ*mptA* (Mishra *et al*., 2007) and processed as described in *Experimental procedures* to afford the double mutant, *C. glutamicum*Δ*mptB*Δ*mptA*. Analysis of this strain showed that its growth characteristics were very similar to *C. glutamicum*Δ*mptB* (data not shown). For further analysis, *C. glutamicum*Δ*mptA*Δ*mptB* was transformed with plasmid-encoded Cg-*mptB*, Cg-*mptA*, Mt-*mptB* and Ms-*mptB*.

### Analysis of lipoglycans from *C. glutamicumΔmptBΔmptA*, *C. glutamicumΔmptBΔmptA* pVWEx-Cg-*mptB* and *C. glutamicumΔmptBΔmptA* pVWEx-Cg-*mptA*

In addition to MptB, *C. glutamicum* possesses the known α(1→6) mannosyltransferase MptA, which is involved in the later stages of core mannan biosynthesis (Mishra *et al*., 2007) and, as a result, we wanted to study the *in situ* specificity of these glycosyltransferases. For this purpose, lipoglycans were extracted from *C. glutamicum*Δ*mptB*Δ*mptA*, and from the same strain carrying either pVWEx-Cg-*mptB* or pVWEx-Cg-*mptA* and analyzed by 15% SDS-PAGE (Fig. 3B). Extracts from *C. glutamicum*Δ*mptB*Δ*mptA* indicated that, as expected, no lipoglycans were present, whereas the presence of pVWEx-Cg-*mptB* resulted in formation of a truncated (Cg-t) version of Cg-LM-A and Cg-LM-B (Mishra *et al*., 2007; 2008). However, lipoglycan extracts from *C. glutamicum*Δ*mptB*Δ*mptA* carrying pVWEx-Cg-*mptA* were identical to that of *C. glutamicum*Δ*mptB*Δ*mptA*, indicating that MptA fails to substitute for MptB in the double mutant. As pVWEx-Cg-*mptA* results in functional MptA (Mishra *et al*., 2007), this result shows that MptA is unable to substitute *in vivo* for MptB. Therefore, both MptA and MptB are distinct and MptB is involved in the initial steps of Cg-LAM, Cg-LM-A and Cg-LM-B biosynthesis, prior to MptA. Furthermore, analysis of *C. glutamicum*Δ*mptB*Δ*mptA* carrying either pVWEx-Mt-*mptB* or pVWEx-Ms-*mptB* resulted in a complete lack of lipoglycan biosynthesis (data not shown), indicating that Mt-MptB and Ms-MptB do not function *in vivo* as the initial α(1→6) mannosyltransferase probably because of an inability to extend Ac_1_PIM_2_ and Man_1_GlcAGroAc_2_ by mannose residues as shown below through *in vitro* chase experiments.

### *In vitro* incorporation of radiolabelled Man from GDP-[^14^C]Man into membrane lipids utilizing *C. glutamicum*, *C. glutamicumΔmptB* and complemented strains

**Fig. 4:**
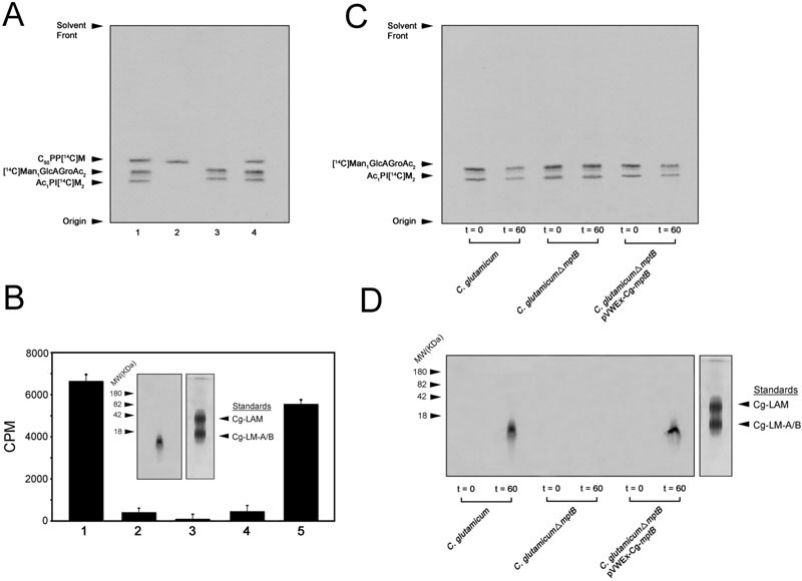
Incorporation of [^14^C]Man from GDP-[^14^C]Man into corynebacterial membrane/cell envelope lipids. A. TLC autoradiography of labelled CHCl_3_/CH_3_OH (2:1)-soluble lipids, C50-PP[^14^C]M, [^14^C]Man_1_GlcAGroAc_2_ and Ac_1_PI[^14^C]M_2_ using GDP-[^14^C]Man and membrane/cell envelope extracts from *C. glutamicum* and *C. glutamicum*Δ*mptB*. Membrane/cell envelope fractions were incubated with GDP-[^14^C]Man in a total volume of 100 μl for 60 min in either the absence or presence of amphomycin (10 μg) and Ca^2+^ ions per reaction mixture pre-incubated with membranes for 15 min. Enzymatically synthesized products C50-PP[^14^C]M, [^14^C]ManGlcAGroAc_2_ and Ac_1_PI[^14^C]M_2_ were isolated as described in *Experimental procedures* to provide washed CHCl_3_/CH_3_OH (2:1)-soluble lipids and also subjected to base treatment. Aliquots (10%) were taken for scintillation counting and the remaining products subjected to TLC/autoradiography using CHCl_3_/CH_3_OH/NH_4_OH/H_2_O (65:25:0.4:3.6, v/v/v/v). *C. glutamicum* CHCl_3_/CH_3_OH (2:1)-soluble lipids (lane 1), base treatment of CHCl_3_/CH_3_OH (2:1)-soluble lipids (lane 2), amphomycin treatment (lane 3) and *C. glutamicum*Δ*mptB* CHCl_3_/CH_3_OH (2:1)-soluble lipids (lane 4). B. Characterization of CHCl_3_/CH_3_OH/H_2_O (10:10:3)-soluble lipids as α(1→6)-linear mannooligosaccharides. The insoluble pellet from the above reaction mixtures following extraction with CHCl_3_/CH_3_OH (2:1) were sequentially washed with 0.9% NaCl in 50% CH_3_OH, 50% CH_3_OH and CH_3_OH, prior to extraction with CHCl_3_/CH_3_OH/H_2_O (10:10:3) and an aliquot (10%) taken for scintillation counting and the remaining product analysed by SDS-PAGE/autoradiography (left-panel inset). *C. glutamicum* (no. 1), amphomycin treatment (no. 2) and acetolysis treatment of CHCl_3_/CH_3_OH/H_2_O (10:10:3)-soluble lipids (no. 3), *C. glutamicum*Δ*mptB* (no. 4) and *C. glutamicum*Δ*mptB* pVWEx-Cg*-mptB* (no. 5) as described in the *Experimental procedures*. C and D. Incorporation of *in vitro in situ* Ac_1_PI[^14^C]M_2_ and [^14^C]Man_1_GlcAGroAc_2_ into α(1→6)-linear mannooligosaccharides with either *C. glutamicum*, *C. glutamicum*Δ*mptB* or *C. glutamicum*Δ*mptB* pVWEx-Cg*-mptB* membrane preparations. Membranes were initially pre-treated with amphomycin, labelled using GDP-[^14^C]Man, re-harvested by centrifugation and extensively washed with buffer. At *t* = 0 min, an aliquot of membranes (20%) was processed as described in the *Experimental procedures* for CHCl_3_/CH_3_OH (2:1)-soluble lipids and analysed by TLC/autoradiography using CHCl_3_/CH_3_OH/NH_4_OH/H_2_O (65:25:0.4:3.6, v/v/v/v) (C) and CHCl_3_/CH_3_OH/H_2_O (10:10:3)-soluble lipids by SDS-PAGE/autoradiography (D). The carefully washed [^14^C]-labelled membranes were re-incubated for a further 60 min following the addition of 0.5 mg cold C50-PPM (Gurcha *et al*., 2002). At *t* = 60 min, an equivalent membrane aliquot as based on *t* = 0 was again analysed for CHCl_3_/CH_3_OH (2:1) and CHCl_3_/CH_3_OH/H_2_O (10:10:3)-soluble lipids as described above (C and D).

Incorporation of [^14^C]Man from GDP-[^14^C]Man into CHCl_3_/CH_3_OH (2:1) and CHCl_3_/CH_3_OH/H_2_O (10:10:3)-soluble lipids was examined using membrane/cell envelope extracts prepared from *C. glutamicum* as described previously utilizing mycobacterial membrane/cell envelope fractions (Besra *et al*., 1997). TLC autoradiography (Fig. 4A, lane 1) of the CHCl_3_/CH_3_OH (2:1)-soluble lipids synthesized by wild-type *C. glutamicum* membrane/cell envelope extracts contained as expected β-D-mannopyranosyl-1-monophosphoryl-decaprenol (C50-PP[^14^C]M), [^14^C]Man_1_GlcAGroAc_2_ and Ac_1_PI[^14^C]M_2_. The identity of the three labelled lipids was established by: (i) base treatment, i.e. degradation of Ac_1_PI[^14^C]M_2_ and [^14^C]Man_1_GlcAGroAc_2_ (Fig. 4A, lane 2), (ii) addition of amphomycin, which specifically chelates polyprenyl phosphates in the presence of Ca^2+^ and thus inhibiting the transfer of Man from GDP-Man to polyprenyl carriers (Fig. 4A, lane 3) and (iii) in comparison with known standards (Tatituri *et al*., 2007). As expected from the analysis of whole cells, *C. glutamicum*Δ*mptB* synthesized comparable levels of all three radiolabelled lipids using membrane/cell envelope extracts prepared from *C. glutamicum*Δ*mptB* (Fig. 4A, lane 4).

The above reaction mixtures were then further processed as described in the *Experimental procedures* section to provide the CHCl_3_/CH_3_OH/H_2_O (10:10:3)-soluble lipids initially using membrane/cell envelope extracts prepared from *C. glutamicum* to provide [^14^C]mannooligosaccharides (Fig. 4B, no. 1), which were further characterized by a series of degradation experiments. The [^14^C]mannooligosaccharides were sensitive to acetolysis (see Fig. 4B, no. 3), thus establishing a core α(1→6)-linear mannan backbone within the CHCl_3_/CH_3_OH/H_2_O (10:10:3)-soluble lipids. In separate experiments, the addition of amphomycin to block C50-PP[^14^C]M synthesis also inhibited the synthesis of the α(1→6)-linear mannan lipids, demonstrating that the synthesis of these CHCl_3_/CH_3_OH/H_2_O (10:10:3)-soluble lipids is PPM-dependent (Fig. 4B, no. 2) and similar to the previously characterized *in vitro* synthesized mycobacterial products (Besra *et al*., 1997). SDS-polyacrylamide gel electrophoresis and subsequent autoradiography of the dried gels demonstrated that the CHCl_3_/CH_3_OH/H_2_O (10:10:3)-soluble lipids (Fig. 4B, no. 1) had slightly reduced mobility, indicating that they were smaller in size (Fig. 4B, left-panel inset), presumably because of their lack of α(1→2) branching characteristic of Cg-LM-A and Cg-LM-B (Tatituri *et al*., 2007). As expected, synthesis of CHCl_3_/CH_3_OH/H_2_O (10:10:3)-soluble lipids using membranes from *C. glutamicum*Δ*mptB* was completely abolished (Fig. 4B, no. 4). Furthermore, complementation with pVWEx-Cg-*mptB* restored synthesis of CHCl_3_/CH_3_OH/H_2_O (10:10:3)-soluble lipids (Fig. 4B, no. 5).

### Chase of *in situ* labelled Ac_1_PI[^14^C]M_2_ and [^14^C]Man_1_GlcAGroAc_2_ into α(1→6)-linear Cg-LM-A and Cg-LM-B utilizing membranes from *C. glutamicum*, *C. glutamicumΔmptB* and *C. glutamicumΔmptB* complemented strains

Amphomycin-treated wild-type *C. glutamicum* membrane/cell envelope extracts were initially pulsed with GDP-[^14^C]Man during a short incubation period (15 min) which was shown earlier to inhibit the synthesis of the CHCl_3_/CH_3_OH/H_2_O (10:10:3)-soluble α(1→6)-linear [^14^C]mannan lipids but, instead of extracting with CHCl_3_/CH_3_OH (2:1), the [^14^C]Man-labelled membranes were re-harvested by ultracentrifugation at 100 000 *g*, carefully washed and re-centrifuged twice using cold buffer, to remove unused GDP-[^14^C]Man. An aliquot of the [^14^C]Man-labelled membranes were extracted with CHCl_3_/CH_3_OH (2:1) and contained as expected solely Ac_1_PI[^14^C]M_2_ (3329 c.p.m.) and [^14^C]Man_1_GlcAGroAc_2_ (5474 c.p.m.) at *t* = 0 chase time as determined by TLC autoradiography and phosphorimaging (Fig. 4C). The CHCl_3_/CH_3_OH/H_2_O (10:10:3)-soluble lipids at *t* = 0 gave 226 c.p.m. The [^14^C]Man-labelled membranes were then further incubated for 60 min following the addition of excess exogenous cold C50-PPM (Gurcha *et al*., 2002) prior to the standard extraction method to provide CHCl_3_/CH_3_OH (2:1) and CHCl_3_/CH_3_OH/H_2_O (10:10:3)-soluble lipids. The *t* = 60 chase time revealed a loss of radioactivity from both Ac_1_PI[^14^C]M_2_ (1709 c.p.m.) and [^14^C]Man_1_GlcAGroAc_2_ (2530 c.p.m.) as determined by TLC autoradiography and phosphorimaging, and incorporation into CHCl_3_/CH_3_OH/H_2_O (10:10:3)-soluble α(1→6)-linear [^14^C]mannooligosaccharide lipids (2895 c.p.m.) (Fig. 4D). The *in vitro in situ* chase experiment demonstrated that the α(1→6)-linear [^14^C]mannooligosaccharide lipids synthesized were elongation products of both Ac_1_PI[^14^C]M_2_ and [^14^C]Man_1_GlcAGroAc_2_. Similar experiments repeated with *C. glutamicum*Δ*mptB in situ* prepared [^14^C]-labelled membranes as above resulted in comparable products at *t* = 0 and *t* = 60 for CHCl_3_/CH_3_OH (2:1)-soluble lipids [Ac_1_PI[^14^C]M_2_ (*t* = 0, 3345 c.p.m.; *t* = 60, 2968 c.p.m.) and [^14^C]Man_1_GlcAGroAc_2_ (*t* = 0, 5840 c.p.m.; *t* = 60, 5025 c.p.m.)] and a lack of the synthesis of α(1→6)-linear [^14^C]mannooligosaccharide lipids (240 c.p.m.) from the elongation primers Ac_1_PI[^14^C]M_2_ and [^14^C]Man_1_GlcAGroAc_2_ following the ‘chase period’ (Fig. 4C and D). Complementation of *C. glutamicum*Δ*mptB* by transformation with plasmid pVWEx-Cg-*mptB* resulted in Ac_1_PI[^14^C]M_2_ (*t* = 0, 3229 c.p.m.; *t* = 60, 1725 c.p.m.) and [^14^C]Man_1_GlcAGroAc_2_ (*t* = 0, 5367 c.p.m.; *t* = 60, 2550 c.p.m.) and *in vitro in situ* synthesis of α(1→6)-linear [^14^C]mannooligosaccharide lipids (2471 c.p.m.) to levels comparable to wild type *C. glutamicum* (Fig. 4C and D). The data clearly demonstrate that Cg-MptB functions *in vivo* and *in vitro* as the initial α-mannosyltransferase, which extends Ac_1_PIM_2_ and Man_1_GlcAGroAc_2_. However, under the same *in vitro in situ* chase conditions, *C. glutamicum*Δ*mptB* pVWEx-Mt-*mptB* (or pVWEx-Ms-*mptB*) failed to elongate the primers Ac_1_PI[^14^C]M_2_ and [^14^C]Man_1_GlcAGroAc_2_ and restore synthesis of the α(1→6)-linear [^14^C]mannooligosaccharides (data not shown). In addition, experiments conducted with *C. glutamicum*Δ*mptB* pVWEx-Mt-*mptB* and *C. glutamicum*Δ*mptB* pVWEx-Ms-*mptB* and the addition of the exogenous primer Ac_1_PI[^14^C]M_4_ isolated from a *M. bovis* BCG PimE mutant also failed to restore the synthesis of the α(1→6)-linear [^14^C]mannooligosaccharides (data not shown).

### *In vitro* analysis of α(1→6) mannosyltransferase activity using *C. glutamicumΔmptB*, *C. glutamicumΔmptBΔmptA* and complemented strains

**Fig. 5:**
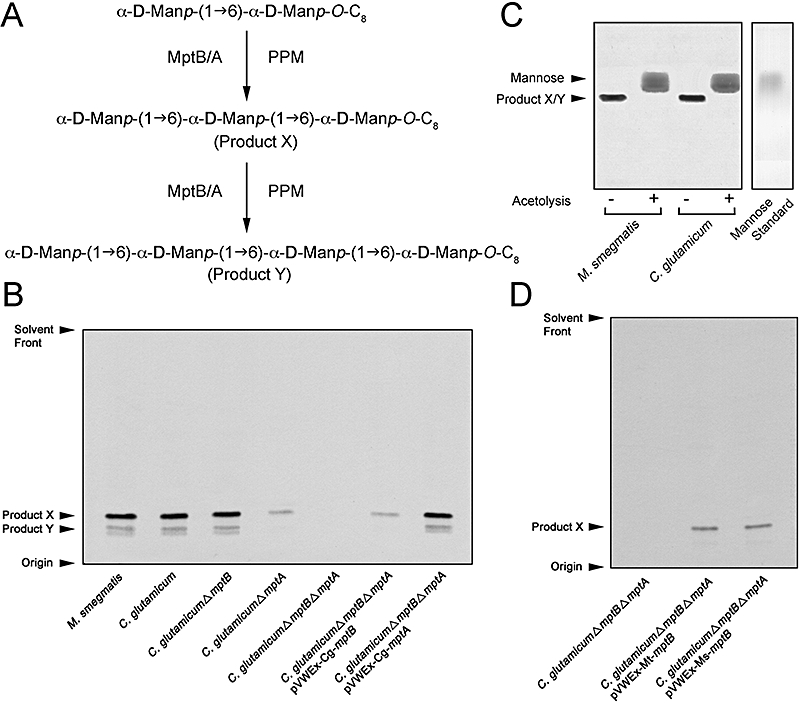
Analysis of products obtained in a cell-free assay for detecting α(1→6) mannosyltransferase activity. A. Biosynthetic reaction scheme of products formed in the α(1→6) mannosyltransferase assay utilizing α-D-Man*p*-(1→6)-α-D-Man*p-O*-C_8_ and C_50_-PP[^14^C]M. B. TLC analysis of products obtained in a cell-free assay for detecting α(1→6) mannosyltransferase activity with membranes prepared from *M. smegmatis*, *C. glutamicum*, *C. glutamicum*Δ*mptB*, *C. glutamicum*Δ*mptA*, *C. glutamicum*Δ*mptB*Δ*mptA*, *C. glutamicum*Δ*mptB*Δ*mptA* pVWEx-Cg*-mptB* and *C. glutamicum*Δ*mptB*Δ*mptA* pVWEx-Cg*-mptA*. C. TLC autoradiography of reaction products X and Y prepared with *M. smegmatis* and *C. glutamicum* membranes and subjected to acetolysis as described in the *Experimental procedures* (Brown *et al*., 1997). D. TLC analysis of products obtained in a cell-free assay for detecting α(1→6) mannosyltransferase activity with membranes prepared from *C. glutamicum*Δ*mptA*Δ*mptB*, *C. glutamicum*Δ*mptA*Δ*mptB* pVWEx-Mt*-mptB* and *C. glutamicum*Δ*mptA*Δ*mptB* pVWEx-Ms*-mptB*. Assays were performed using the synthetic α-D-Man*p*-(1→6)-α-D-Man*p-O*-C_8_ neoglycolipid acceptor in a cell-free assay as described (Brown *et al*., 2001). The products of the assay were re-suspended in *n*-butanol before scintillation counting. The incorporation of [^14^C]Man*p* was determined by subtracting counts present in control assays (incubations in the absence of acceptor), which were typically less than 100 c.p.m. per assay. The remaining labelled material was subjected to TLC using silica gel plates (5735 silca gel 60F254, Merck) developed in CHCl_3_:CH_3_OH:H_2_O; NH_4_OH (65:25:3.6:0.5, v/v/v/v) and the products visualized by phosphorimaging (Kodak K Screen). The results represent triplicate assays in three independent experiments. A schematic representation of the reaction is showed in (A) and the products X and Y are indicated by arrows.

Initial attempts to develop an *in vitro* assay using either purified recombinant-expressed MptB, or *Escherichia coli* membranes harbouring the protein, have thus far proved unsuccessful. Alternatively, we assessed the capacity of membrane preparations from *C. glutamicum* and its recombinant strains to catalyse α(1→6) mannosyltransferase activity in a previously defined acceptor assay utilizing the neoglycolipid acceptor α-D-Man*p*-(1→6)-α-D-Man*p*-O-C_8_ and C_50_-PP[^14^C]M as a sugar donor (Brown *et al*., 2001) (Fig. 5A). The TLC autoradiography of products from *in vitro* assays when assayed with wild-type *C. glutamicum* resulted in the formation of product X, a trisaccharide α-D-[^14^C]Man*p*-(1→6)-α-D-Man*p*-(1→6)-α-D-Manp-O-C_8_, and product Y, a tetrasaccharide α-D-[^14^C]Man*p*-(1→6)-α-D-Man*p*-(1→6)-α-D-Man*p*-(1→6)-α-D-Manp-O-C_8_ (Fig. 5B). These products comigrated on TLC autoradiography with the corresponding products previously chemically characterized and prepared using mycobacterial membranes, and were cleaved by acetolysis, demonstrating that they were α(1→6)-linked [^14^C]Man products (Fig. 5B and C) (Brown *et al*., 1997; 2001). The intensity of the major product X, a trisaccharide α-D-[^14^C]Man*p*-(1→6)-α-D-Man*p*-(1→6)-α-D-Manp-O-C_8_, was consistently slightly reduced in the case of *C. glutamicum*Δ*mptB* (89 217 ± 4269 c.p.m.) in comparison with wild-type *C. glutamicum* (92 325 ± 5017 c.p.m.) (Fig. 5B). This reduction in activity corresponded to the residual α(1→6) mannosyltransferase activity observed in *C. glutamicum*Δ*mptA* (2053 ± 604 c.p.m.) (Fig. 5B) (Mishra *et al*., 2007). These results suggested the presence of two α(1→6) mannosyltransferase activities utilizing this neoglycolipid acceptor, catalysed by MptA and MptB, with the former more efficiently utilizing the neoglycolipid acceptor as a substrate. Assays containing membrane preparations from *C. glutamicum*Δ*mptB*Δ*mptA* showed no product formation on TLC, indicating a complete abrogation of both α(1→6) mannopyranosyltransferase activities from *C. glutamicum* (Fig. 5B). Analysis of the double mutant with pVWEx-Cg-*mptB* revealed a significant but weak band (2682 ± 940 c.p.m.) corresponding to product X on TLC analysis; however, when complemented with pVWEx-Cg-*mptA*, a similar phenotype to that of *C. glutamicum*Δ*mptB* could be observed (80 614 ± 4135 c.p.m. for X), although at a slower transfer rate. The data confirmed that NCgl1505 is an α(1→6) mannopyranosyltransferase; however, the specific α(1→6) mannopyranosyltransferase activity is much lower in comparison with MptA, under the assay conditions utilizing the neoglycolipid acceptor.

### *In vitro* and mutational analysis of the mycobacterial MptB

**Fig. 6:**
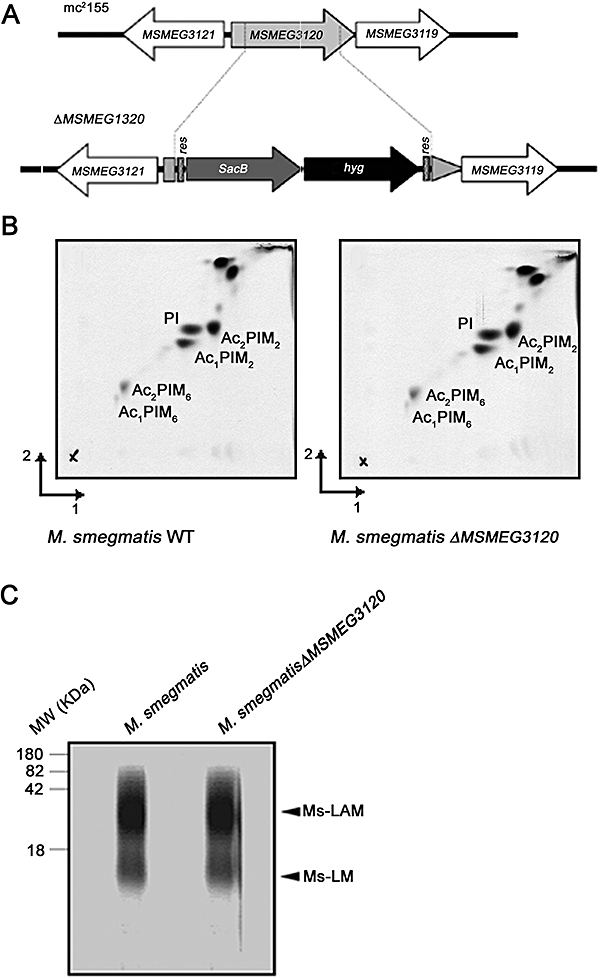
Characterization of a *M. smegmatis mptB* (*MSMEG3120*) mutant. A. Map of the *MSMEG3120* region in the wild type, parental strain *M. smegmatis* mc^2^155 and its corresponding region in the Δ*MSMEG3120* mutant. *res*, resolvase site; *hyg*, hygromycin-resistance gene from *Streptomyces hygroscopicus*; *sacB*, sucrose counterselectable gene from *Bacillus subtilis*. B. 2D-TLC analysis of the [^14^C]-labelled (50 000 c.p.m.) polar lipids fraction from *M. smegmatis* (WT) and *M. smegmatis*Δ*MSMEG3120* strains. The polar lipid extract was examined on aluminum-backed plates of silica gel 60 F254 (Merck 5554), using CHCl_3_/CH_3_OH/H_2_O (60:30:6, v/v/v) in the first direction and CHCl_3_/CH_3_COOH/CH_3_OH/H_2_O (40:25:3:6, v/v/v/v) in the second direction. Lipids were visualized by autoradiography by overnight exposure of Kodak X-Omat AR film to the TLC plates to reveal labelled lipids. C. Lipoglycan analysis of wild-type *M. smegmatis* and *M. smegmatis*Δ*MSMEG3120* using SDS-PAGE and visualized using a Pro-Q emerald glycoprotein stain (Invitrogen). The four major bands represent glycoproteins of 180, 82, 42 and18 kDa respectively.

To study the function of the mycobacterial MptB, we transformed the *C. glutamicum*Δ*mptB*Δ*mptA* double mutant with a plasmid containing either *M. tuberculosis Rv1459c* (pVWEx-Mt-*mptB*) or *M. smegmatis MSMEG3120* (pVWEx-Ms-*mptB*). Membrane preparations of these strains restored *in vitro*α(1→6) mannopyranosyltransferase activity (Fig. 5D) by formation of the trisaccharide product X (Mt-MptB, 3159 ± 456 c.p.m. and Ms-MptB, 2949 ± 378 c.p.m.) to a similar level to that of the isogenic strain with pVWEx-Cg-*mptB* (Fig. 5B), showing that the *M. tuberculosis* and *M. smegmatis* gene could restore activity in an *in vitro* cell-free assay with the *C. glutamicum* double mutant. We then generated a null mutant of *M. smegmatis* mc^2^155 *MSMEG3120* (homologue of *Rv1459c*) using specialized transduction (Fig. 6A), and analysed total lipids and lipoglycans in the mutant strain. Surprisingly, the mutant strain Δ*MSMEG3120* had a total lipid profile identical to the parental wild-type strain *M. smegmatis* mc^2^155 (TLC system designed to separate PIMs and other phospholipids is shown in Fig. 6B) and also synthesized LM and LAM (Fig. 6C). These results suggested that MSMEG3120, unlike its corynebacterial counterpart, was redundant and it was likely that another α-mannosyltransferase compensated for the loss of its function in the Δ*MSMEG3120* mutant.

## Discussion

**Fig. 7:**
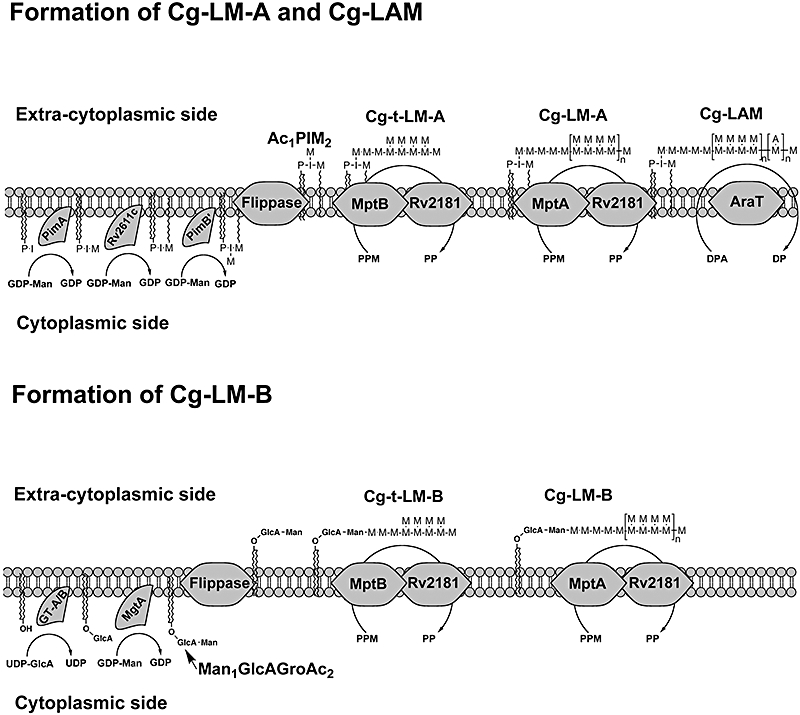
Schematic representation of the glycosyltransferases involved in *C. glutamicum* lipoglycan biosynthesis.

Over the past decade, much research has been carried out on the mechanisms and genetics of mycobacterial cell wall carbohydrate biosynthesis, particularly the formation of the essential AG (Daffe *et al*., 1993; Besra *et al*., 1995; Belanger *et al*., 1996; Kremer *et al*., 2001; Alderwick *et al*., 2005; 2006a, 2006b; Berg *et al*., 2007; Seidel *et al*., 2007) and the immunomodulatory heteropolysaccharides LM and LAM (Schaeffer *et al*., 1999; Kordulakova *et al*., 2002; Kremer *et al*., 2002; Zhang *et al*., 2003; Dinadayala *et al*., 2006; Kaur *et al*., 2006; 2007; Mishra *et al*., 2007). An archetypal biosynthetic pathway is now emerging for the formation of these important macromolecules, which predominantly include enzymes from the GT-A, B and C superfamily of glycosyltransferases (Liu and Mushegian, 2003) (Fig. 1). PimA, PimB, PimB′ and PimC, all of which are GT-A/B glycosyltransferases, have been shown to be involved in PIM biosynthesis, which serves as a substrate for LM/LAM extension and maturation (Schaeffer *et al*., 1999; Kordulakova *et al*., 2002; Kremer *et al*., 2002; Lea-Smith *et al*., 2008; Mishra *et al*., 2008). We and others recently identified the GT-C glycosyltransferase MptA as an α(1→6) mannosyltransferase involved in intermediate LM biosynthesis, specifically in distal α(1→6) core LM formation (Kaur *et al*., 2007; Mishra *et al*., 2007). Apart from a core α(1→6) mannan backbone, α(1→2) mannose residues punctuate LM, and the GT-C glycosyltransferase Rv2181 has been identified to be responsible for some, if not all, of these branched mannose residues (Kaur *et al*., 2006). At some point, LM is further glycosylated by other GT-C glycosyltransferases, such as EmbC for the biosynthesis of LAM (Zhang *et al*., 2003) and then mannose-capped (Dinadayala *et al*., 2006; Appelmelk *et al*., 2007). In this study, we have characterized the role of a putative glycosyltransferase (NCgl1505) belonging to the GT-C superfamily of glycosyltransferases (Liu and Mushegian, 2003) by virtue of genomic deletion in *C. glutamicum*. We present MptB as a PPM-dependent α(1→6) mannosyltransferase, involved in early stages of proximal α(1→6) core Cg-LM-A and Cg-LM-B biosynthesis in *C. glutamicum* (Fig. 7).

Our initial *in vivo* and *in vitro* studies of PIM and Man_1_GlcAGroAc_2_ biosynthesis in *C. glutamicum*Δ*mptB* highlighted no apparent change in lipid profiles, compared with those from wild-type *C. glutamicum* (Figs S1 and 4A). It is reasonable to conclude from the data that MptB is not involved in either early PIM or Man_1_GlcAGroAc_2_ biosynthesis. This was not surprising as these early biosynthetic steps are completely unique to enzymes belonging to the GT-A/B glycosyltransferase family, which utilize GDP-mannose as a substrate (Liu and Mushegian, 2003). Assays utilizing membrane preparations from *C. glutamicum* and *C. glutamicum*Δ*mptB* indicated that there was no further accumulation of higher mannosylated versions of PIMs and Man_1_GlcAGroAc_2_. The lack of higher mannosylated versions in *C. glutamicum* suggests that the next committed step in lipoglycan biosynthesis stems from Ac_1_PIM_2_ and Man_1_GlcAGroAc_2_ and that this is catalysed by Cg-MptB.

As a result of absence of MptB, *C. glutamicum*Δ*mptB* is unable to synthesize Cg-LAM, Cg-LM-A and Cg-LM-B *in vivo*, which is in contrast to our earlier studies on MptA, where a truncated Cg-LM-A and Cg-LM-B species was synthesized (Mishra *et al*., 2007). In *C. glutamicum*, we now also present *in vitro* evidence that Ac_1_PIM_2_ and Man_1_GlcAGroAc_2_ are acceptors for Cg-MptB, the first GT-C α-mannosyltransferase committed to Cg-LM-A and Cg-LM-B biosynthesis. This is supported by *in vitro in situ* chase experiments elongating the Ac_1_PI[^14^C]M_2_ and [^14^C]Man_1_GlcAGroAc_2_ primers by the sugar donor C50-PPM. These crucial observations, together with the presence of Ac_1_PIM_2_ and Man_1_GlcAGroAc_2_, completely support our hypothesis that Cg-MptB mannosylates Ac_1_PIM_2_ and Man_1_GlcAGroAc_2_. Our previous experiments on glycosyltransferase activities in membranes prepared from *C. glutamicum*Δ*mptA* identified a residual α(1→6) mannosyltransferase activity (Mishra *et al*., 2007). This α-mannosyltransferase activity can now be attributed to the presence of MptB as, upon its deletion in *C. glutamicum*, a partial depletion in α(1→6) mannosyltransferase activity is observed and a complete loss of activity is found upon deletion of both Cg-*mptA* and Cg-*mptB*. These data together with the *in vivo* analyses identify MptB as a *bona fide*α(1→6) mannosyltransferase. Interestingly, α(1→6) mannan extension is more complex in *Mycobacterium* based on the evidence that Mt-MptB and Ms-MptB fail to complement the *C. glutamicum*Δ*mptB* mutant and suggests a slightly different substrate specificity of the MptB orthologues of *M. tuberculosis* and *M. smegmatis*. Although, clearly α(1→6) mannosyltransferase(s) based on *in vitro* data, studies are currently underway exploring heterologous protein expression systems for Mt-MptB and Ms-MptB in combination with a variety of substrates in a revised *in vitro* assay format.

Given the high degree of homology between the *C. glutamicum* and mycobacterial orthologues of MptB and the similar organization of neighbouring genes in the two genera, we expected deletion of *M. smegmatis mptB* (*MSMEG3120*) to have the same effect as that in *C. glutamicum*. However, surprisingly, the *M. smegmatis mptB* mutant still synthesised LM and LAM, indicating that another, yet unidentified, α-mannosyltransferase could substitute for MptB in the mutant *M. smegmatis* strain. It has been previously shown that a high degree of functional redundancy exists in key enzymes involved in mycobacterial cell wall assembly, for instance, PimB/PimB′ and MgtA (Schaeffer *et al*., 1999; Tatituri *et al*., 2007; Lea-Smith *et al*., 2008; Mishra *et al*., 2008), PimC (Kremer *et al*., 2002), and EmbA and EmbB (Berg *et al*., 2007) in PIM/LM/LAM and AG biosynthesis, and the antigen 85 complex in mycolic acid biosynthesis (Puech *et al*., 2002). In this particular case, the *C. glutamicum* mutant study enabled the assignment of function to the GT-C glycosyltransferase NCgl1505, which would have otherwise not been possible if similar studies would have concentrated solely on mycobacterial species.

Interestingly, the mechanism of how Ac_1_PIM_2_ traverses the cytoplasmic membrane remains poorly understood. Bioinformatic inspection of the locus surrounding MptB has highlighted two possible candidates for potential flippases. Downstream of the putative glycosyltransferase Rv1459c, three conserved genes are located in all *Corynebacterinaeae* and the expression of the four-gene locus in *C. glutamicum* is translationally coupled (Wang *et al*., 2006). This presents strong evidence for a functional coupling of the putative glycosyltransferase Rv1459c with Rv1458c, Rv1457c and Rv1456c. The latter genes encode for two ABC transporter integral membrane proteins, with Rv1458c encoding for an ATP-dependent binding protein. Applying structure prediction comparisons and hidden Markov models (Soding *et al*., 2005), Rv1458c exhibits remote structural similarities to sugar-binding proteins of ABC carriers, such as the sugar-binding protein of *Pyrococcus horikoshii* or the maltose/maltodextrin-binding protein MALK of *E. coli* (Lu *et al*., 2005). Rv1457c encodes a permease component of an ABC-2-type transporter, characteristically involved in catalysing the export of drugs and carbohydrates (Reizer *et al*., 1992). As transmembrane channels of ABC-2-type transporters are either homo- or heterooligomers and Rv1456c has features of a transporter protein, it is plausible to suggest that the membrane channel coupled to the glycosyltransferase might be a heterooligomer made up of Rv1457c and Rv1456c. In a previous study, Wang *et al*. (2006) proposed that one or more of the proteins encoded by the orthologues of Rv1456c-Rv1459c gene locus in *C. matruchotii* was involved in mycolic acid transport. A transposon mutant with an insertion in the cluster had an altered mycolic acid profile. However, in light of the evidence described in this work, this change in mycolylation may be an indirect effect as a result of the loss of Cg-LAM and Cg-LM-A/B. Further examination of this gene locus is required for characterization of potential roles in mycolic acid and glycolipid transport across the membrane bilayer.

## Experimental procedures

### Bacterial strains and growth conditions

*Corynebacterium glutamicum* ATCC 13032 (referred to the remainder of the text as *C. glutamicum*) and *E. coli* DH5αmcr were grown in Luria–Bertani broth (Difco) at 30°C and 37°C respectively. The recombinant strains generated in this study were grown on rich BHI medium (Difco), and the salt medium CGXII used for *C. glutamicum* as described (Eggeling and Bott, 2005). Kanamycin and ampicillin were used at a concentration of 50 μg ml^−1^. Samples for lipid analysis were prepared by harvesting cells at an OD of 10–15, followed by a saline wash and freeze drying. *M. smegmatis* strains were grown in Tryptic Soy Broth (TSB; Difco) containing 0.05% Tween80 (TSBT). Solid media were made by adding 1.5% agar to the above-mentioned broths. The concentrations of antibiotics used for *M. smegmatis* were 100 μg ml^−1^ for hygromycin and 20 μg ml^−1^ for kanamycin. *M. tuberculosis* H37Rv DNA was obtained from the NIH Tuberculosis Research Materials and Vaccine Testing Contract at Colorado State University. All other chemicals were of reagent grade and obtained from Sigma-Aldrich.

### Construction of plasmids and strains

The genes analysed were the orthologues of *Rv1459c* and *NCgl1505* from *M. tuberculosis* and *C. glutamicum*, respectively, termed *mptB*. The vectors made were pVWEx-Mt-*mptB*, pVWEx-Ms-*mptB*, pVWEx-Cg-*mptB*, pET-Mt-*mptB*, pET-Cg-*mptB* and pK19mobsacBΔ*mptB*. To construct the deletion vector pK19mobsacBΔ*mptB*, cross-over PCR was applied with primer pairs AB (A, CGTTAAGCTTCCAAAGGTAACCTTATTTATGCTGGCCACAGG; B, CCCATCCACTAAACTTAAACACGATGCGCGGCAAAGT) and CD (C, TGTTTAAGTTTAGTGGATGGGGAGTTTGAGGCGGAATCC; D, GCATGGATCCGCGGTAAAACCTTCGCACATTTCAATG) (all primers in 5′-3′ direction) and *C. glutamicum* genomic DNA as template. Both amplified products were used in a second PCR with primer pairs AD to generate a 597 bp fragment consisting of sequences adjacent to Cg-*mptB*, which was ligated with HindIII-BamHI-cleaved pK19mobsacB.

To enable expression of Cg-*mptB* in *C. glutamicum*, the primer pair EF was used (E, CGAATTGGATCCTCAGTGTAAACCAAAGGTTGGATTCC; F, GATATGTTAACAGGGAGATATAGTTGCCGCGCATCGG) to amplify *C. glutamicum mptB*, which was ligated with SalI-, BamHI-cleaved pVWEx to generate pVWEx-Cg-*mptB*. For expression of Cg-*mptB* in *E. coli*, the primer pair GF was used (G, CGCGTCATATGTTGCCGCGCATCGGCAC) and the resulting product ligated with NdeI-, BamHI-cleaved pET16b (Novagen) to generate pET-Cg-*mptB*. To enable expression of Mt-*mptB* in *C. glutamicum*, the primer pair HJ was used (H, GATATGTTAACAGGGAGATATAGATGGCAGCCCGCCAC; J, GGAATTGGATCCTCACGTGGAATCAGCGTAGGCG) to amplify *M. tuberculosis mptB*, which was ligated with SalI-, BamHI-cleaved pVWEx to generate pVWEx-Mt-*mptB*. To express Mt-*mptB* in *E. coli*, the primer pair JK (K, CTTAATGGATCCATGGCAGCCCGCCAC) was used and the resulting product ligated with BamHI-cleaved pET16b to generate pET-Mt-*mptB*. To enable expression of Ms-*mptB* in *C. glutamicum*, the primer pair MsB_for (5′-CGCGTCGACAAGGAGATATAGATATGATGGCCAGCCGCCTGTCGT-3′) and MsB_rev (5′-CCGGAATTCTTACGGGGATTCAGCGTAGGCGTC-3′) was used to amplify Ms*-mptB*, which was cloned in pUC18 and ligated as an EcoRI/SalI fragment with similar cleaved pEKEx2 to generate pEKEx2-Ms-*mptB*.

All plasmids were confirmed by sequencing. The chromosomal deletion of Cg-*mptB* was performed as described previously using two rounds of positive selection (Schafer *et al*., 1994), and its successful deletion was verified by use of primer pair AB and the additional primer pair LM (L, GCGCGTATCACCGTCTCCGGTGTG; M, GCTGTTGGCCACCTGACAGACGTCG). Because of the similarity of MptB with MptA, *C. glutamicum*Δ*mptB* was transformed together with pK19mobsacBΔ*mptA* (Mishra *et al*., 2007) to yield the double mutant *C. glutamicum*Δ*mptB*Δ*mptA*. Plasmids pVWEx-Mt-*mptB*, pVWEx-Ms-*mptB* and pVWEx-Cg-*mptB* were introduced into *C. glutamicum*Δ*mptB* and *C. glutamicum*Δ*mptB*Δ*mptA* by electroporation with selection to kanamycin resistance (25 μg ml^−1^).

To generate an allelic recombination substrate to replace *MSMEG3120* with *hyg*, approximately 1 kb of upstream and downstream flanking sequences were PCR-amplified from *M. smegmatis* mc^2^155 genomic DNA using the primer pairs MS3120LL (ttt-ttt-ttc-cat-aaa-ttg gAT-TGT-GAC-GGA-ATT-CGT-CCG-ACG-GT) and MS3120LR (ttt-ttt-ttc-cat-ttc-ttg-gAT GCC-CTG-ACC-GAT-CCA-CAG-GAA), and MS3120RL (ttt-ttt-ttc-cat-aga-ttg-gTG-TTC-CAG-ATC-GTC-ATG-GCA-ACC-CT) and MS3120RR (ttt-ttt-ttc-cat-ctt-ttg-gAT-GAT-CAC-GAT-GCG-ATC-GGC-GAG-TT) respectively. The PCR products consisted of a 682 bp upstream DNA fragment (including the last 15 bp coding sequence of *MSMEG3121*, 89 bp intergenic sequence and the first 578 bp of *MSMEG3120*) and a 813 bp downstream DNA fragment (including the last 160 bp of *MSMEG3120* and the first 655 bp of *MEMEG3119*). Following restriction digestion of the primer-introduced *Van*91I sites (shown in lower case), the PCR fragments were cloned into *Van*91I-digested p0004S to yield the knockout plasmid pΔ*MSMEG3120* which was then packaged into the temperature-sensitive mycobacteriophage phAE159 as described previously (Bardarov *et al*., 2002), to create a recombinant phage, which was then used to transduce wild-type *M. smegmatis* mc^2^155 to generate the Δ*MSMEG3120* deletion mutant which was confirmed by Southern blot and PCR analysis (data not shown).

### Lipid extraction and analysis

Polar lipids and apolar lipids were extracted as described previously (Dobson *et al*., 1985). Briefly, 6 g of dried cells of wild-type, mutant and complemented strains of *C. glutamicum* or *M. smegmatis* were mixed thoroughly using the biphasic mixture of methanolic saline (220 ml containing 20 ml of 0.3% NaCl and 200 ml of CH_3_OH) and petroleum ether (220 ml) for 2 h. The upper petroleum-ether layer containing apolar lipids were separated following centrifugation. The lower methanolic saline extract was further extracted using petroleum ether (220 ml), mixed and centrifuged. The two upper petroleum-ether fractions were combined and dried. Polar lipids were extracted by the addition of CHCl_3_/CH_3_OH/0.3% NaCl (260 ml, 9:10:3, v/v/v) added to the lower methanolic saline phase and stirred for 4 h. The mixture was filtered and the filter cake re-extracted twice with CHCl_3_/CH_3_OH/0.3% NaCl (85 ml, 5:10:4, v/v/v). CHCl_3_ (145 ml) and 0.3% NaCl (145 ml) were added to the combined filtrates and stirred for 1 h. The mixture was allowed to settle, and the lower layer containing the polar lipids recovered and dried. The polar lipid extract was examined by 2D-TLC on aluminum-backed plates of silica gel 60 F254 (Merck 5554), using CHCl_3_/CH_3_OH/H_2_O (60:30:6, v/v/v) in the first direction and CHCl_3_/CH_3_COOH/CH_3_OH/H_2_O (40:25:3:6, v/v/v/v) in the second direction. *C. glutamicum* glycolipids were visualized by either spraying plates with α-naphthol/sulphuric acid or 5% ethanolic molybdophosphoric acid followed by gentle charring of plates. Identification of phospholipids was carried out using the Dittmer and Lester reagent as described (Tatituri *et al*., 2007).

### Extraction and purification of lipoglycans

Lipoglycans from *C. glutamicum* and *M. smegmatis* strains were extracted as described previously (Nigou *et al*., 1997; Ludwiczak *et al*., 2002). Briefly, delipidated cells were re-suspended in deionized water and disrupted by probe sonication (MSE Soniprep 150, 12 μm amplitude, 60 s on, 90 s off for 10 cycles, on ice). Ethanol extraction was carried out by mixing C_2_H_5_OH/H_2_O (100 ml, 1:1, v/v) to the cell suspension and refluxing at 68°C, for 12 h intervals, followed by centrifugation and recovery of the supernatant. This C_2_H_5_OH/H_2_O extraction process was repeated five times and the combined supernatants dried. The dried supernatant was then treated with phenol/H_2_O (80%, w/w) at 70°C for 1 h followed by dialysis using a 1500 MWCO membrane (Spectrapore) against deionized water. The retentate was dried, re-suspended in water and treated sequentially digested with α-amylase, DNase, RNase chymotrypsin and trypsin, and the lipoglycan recovered following extensive dialysis using a 1500 MWCO membrane (Spectrapore) against deionized water (Nigou *et al*., 1999). The lipoglycans were monitored on 15% SDS-PAGE using either a silver stain utilizing periodic acid and silver nitrate (Hunter *et al*., 1986) or a Pro-Q emerald glycoprotein stain (Invitrogen).

### Extraction and analysis of [^14^C]PIMs from *M. smegmatis* strains

*Mycobacterium smegmatis* cultures (5 ml) were grown in TSB and metabolically labelled using 1 μCi ml^−1^[1,2-^14^C]acetate (50–62 mCi mmol^−1^, GE Healthcare, Amersham Bioscience) at an OD600 of 0.4 and cultures grown for a further 4 h at 37°C with gentle shaking. Cells were harvested by centrifugation, washed once with PBS and a small-scale apolar and polar lipid extraction performed according to the methods of Dobson *et al*. (1985). The polar lipid extracts were re-suspended in CHCl_3_:CH_3_OH (2:1) and crude lipid (50 000 c.p.m.) applied to the corners of 6.6 × 6.6 cm pieces of Merck 5554 aluminium-backed TLC plates. The plates were developed using CHCl_3_:CH_3_OH:H_2_O (60:30:6, v/v/v) in the first direction and CHCl_3_:CH_3_COOH:CH_3_OH:H_2_O (40:25:3:6, v/v/v/v) in the second direction to separate [^14^C]-labelled PIMs. Lipids were visualized by autoradiography by overnight exposure of Kodak X-Omat AR film to the TLC plates to reveal labelled lipids and compared with known standards.

### Preparation of enzymatically active membranes and cell envelope fraction

*Mycobacterium smegmatis and C. glutamicum* strains used in this study were cultured to the mid-logarithmic growth phase in 1 l BHIS medium supplemented with kanamycin (25 μg ml^−1^) and IPTG (0.2 mM) where appropriate. Cells were harvested by centrifugation, re-suspended in 20 ml of buffer A (50 mM MOPS pH 7.9, 5 mM β-mercaptoethanol and 5 mM MgCl_2_) and lysed immediately by sonication (60 s on, 90 s off for a total of 10 cycles). The lysate was clarified by centrifugation at 27 000 *g* (4°C, 30 min) and membranes were deposited by centrifugation of the supernatant at 100 000 *g* (4°C, 90 min). The membranes were re-suspended in buffer A to a final protein concentration of 20 mg ml^−1^. The 27 000 *g* pellet was re-suspended in 10 ml of buffer A and 15 ml of Percoll (Pharmacia, Sweden), and centrifuged at 27 000 *g* for 60 min at 4°C. The particulate, upper diffuse band, containing both cell walls and membranes, was removed, collected by centrifugation, washed three times in buffer A, and finally re-suspended in 1 ml of buffer A. The final concentration of this Percoll-60 cell envelope fraction (P-60) was 20 mg ml^−1^.

### *In vitro* incorporation of radiolabelled Man from GDP-[^14^C]Man into membrane lipids

Initial assays involved incubation of membranes (0.5 mg of protein), P-60 fraction (0.5 mg of protein) in buffer A, containing 1 mM ATP and 0.25 μCi of GDP-[^14^C]Man (Amersham Pharmica Biotech, Uppsala, Sweden, 303 mCi mmol^−1^) in a final volume of 100 μl incubated at 37°C for 60 min as described (Besra *et al*., 1997). The reactions were terminated by the addition of CHCl_3_/CH_3_OH (6 ml, 2:1, v/v), centrifuged and the pellet re-extracted thrice using CHCl_3_/CH_3_OH (6 ml, 2:1, v/v). The resulting insoluble pellet was sequentially washed three times with 0.9% NaCl in CH_3_OH (2 ml), CH_3_OH/H_2_O (2 ml, 1:1, v/v) and CH_3_OH (2 ml) to remove residual GDP-[^14^C]Man before extracting three times with CHCl_3_/CH_3_OH/H_2_O (2 ml, 10:10:3, v/v/v). The CHCl_3_/CH_3_OH/H_2_O (10:10:3)-soluble lipids were dried and re-suspended in 200 μl of CHCl_3_/CH_3_OH/H_2_O (10:10:3, v/v/v) and an aliquot (10%) of the resulting [^14^C]-labelled mannooligosaccharide polymers [α(1→6)-linear-LM-A and α(1→6)-linear-LM-B] quantified by liquid scintillation counting using 5 ml of EcoScintA (National Diagnostics, Atlanta, GA). The remaining aliquot was analysed by SDS-PAGE/autoradiography. The original combined CHCl_3_/CH_3_OH (2:1) organic extracts were dried and re-suspended in CHCl_3_/CH_3_OH/H_2_O (4 ml, 10:10:3, v/v/v) followed by the addition of 1.75 ml of CHCl_3_ (1.75 ml) and H_2_O (0.75 ml). The reaction mixture was vortexed, centrifuged and the upper aqueous phase removed. The organic phase was washed three times with CHCl_3_/CH_3_OH/H_2_O (2 ml, 3:47:48, v/v/v), and the final organic extract dried under a stream of nitrogen to afford C50-PP[^14^C]M, Ac_1_PI[^14^C]M_2_ and [^14^C]Man_1_GlcAGroAc_2_. Alternatively, the combined CHCl_3_/CH_3_OH (2:1) organic extracts were dried and re-suspended in CHCl_3_/CH_3_OH/0.8 M NaOH (4 ml, 10:10:3, v/v/v) and heated at 50°C for 30 min, followed by the addition of CHCl_3_ (1.75 ml) and H_2_O (0.75 ml), and processed as described above to afford C50-PP[^14^C]M. The resulting C50-PP[^14^C]M, Ac_1_PI[^14^C]M_2_ and [^14^C]Man_1_GlcAGroAc_2_ products were subjected to TLC/autoradiography using silica gel plates (5735 silca gel 60F254, Merck) developed in CHCl_3_:CH_3_OH:H_2_O:NH_4_OH (65:25:3.6:0.5, v/v/v/v) and the products visualized and quantified by phosphorimaging (Kodak K Screen).

### Pre-treatment of membranes with amphomycin and further incorporation of *in situ* labelled [^14^C]Man-labelled membrane glycolipids into CHCl_3_/CH_3_OH/H_2_O (10:10:3)-soluble [^14^C]-labelled mannooligosaccharide polymers

The lipopetide amphomycin (2 mg) was dissolved in 500 μl of 0.1 M acetic acid, and the solution adjusted to 0.05 M sodium acetate (pH 7.0) with 0.1 M NaOH for a final concentration of 2 mg ml^−1^ (Gurcha *et al*., 2002). Membranes/cell envelope (5 mg) in 500 μl of buffer A were pre-incubated with amphomycin (10 μg per 100 μl reaction mixture) at 37°C for 15 min, resulting in inhibition of PPM synthesis, prior to a further short 15 min pulse incubation with 1.25 μCi of GDP-[^14^C]Man (Amersham Pharmica Biotech, Uppsala, Sweden, 303 mCi mmol^−1^). A 20% aliquot of the reaction mixture was processed as described above to afford Ac_1_PI[^14^C]M_2_, [^14^C]Man_1_GlcAGroAc_2_ and CHCl_3_/CH_3_OH/H_2_O (10:10:3)-soluble lipids. The remaining amphomycin-treated membranes/cell envelope containing *in situ*[^14^C]Man-labelled lipids (Ac_1_PIM_2_ and Man_1_GlcAGroAc_2_) were diluted with buffer A and recovered by re-centrifugation at 100 000 *g* for 60 min, carefully washed and re-centrifuged with cold buffer A twice, thereby ensuring complete removing of unused GDP-[^14^C]Man (Besra *et al*., 1997). The [^14^C]Man-labelled membranes were then carefully re-suspended in 400 μl buffer A prior to the addition of 0.5 mg C50-PPM in 1% IgePal CA-630 (40 μl, Sigma Aldrich) (Gurcha *et al*., 2002), incubated further at 37°C for 60 min and a 100 μl aliquot processed/analysed as described above to provide the CHCl_3_/CH_3_OH (2:1) and CHCl_3_/CH_3_OH/H_2_O (10:10:3)-soluble [^14^C]-labelled mannose-containing products.

### *In vitro* analysis of α(1→6) mannosyltransferase activity

The neoglycolipid acceptors α-D-Man*p*-(1→6)-α-D-Man*p*-O-C_8_ (stored in C_2_H_5_OH) and C_50_-PP[^14^C]M (stored in CHCl_3_/CH_3_OH, 2:1, v/v), prepared as described (Gurcha *et al*., 2002), were separated into aliquots into 1.5 ml eppendorf tubes to a final concentration of 2 mM and 0.25 μCi (0.305 Ci mmol^−1^) respectively, and dried under nitrogen. IgePal CA-630 (8 μl, Sigma Aldrich) was added and the tubes sonicated to re-suspend the lipid-linked components, and the remaining assay components in a final volume of 80 μl were added, which included: 1 mM ATP, 1 mM NADP, and membrane protein (1 mg) from either *C. glutamicum*, *C. glutamicum*Δ*mptB*, *C. glutamicum*Δ*mptA*, *C. glutamicum*Δ*mptB* pVWEx-Cg-*mptB*, *C. glutamicum*Δ*mptB*Δ*mptA*, *C. glutamicum*Δ*mptB*Δ*mptA* pVWEx-Cg-*mptB*, *C. glutamicum*Δ*mptB*Δ*mptA* pVWEx-Cg-*mptA*, *C. glutamicum*Δ*mptB*Δ*mptA* pVWEx-Mt-*mptB* and *C. glutamicum*Δ*mptB*Δ*mptA* pVWEx-Ms-*mptB.* Assays were incubated at 37°C for 1 h and then quenched by the addition of CHCl_3_/CH_3_OH (533 μl, 1:1, v/v). The reaction mixtures were then centrifuged at 27 000 *g* for 15 min at 4°C, the supernatant removed and dried under nitrogen. The residue was re-suspended in C_2_H_5_OH/H_2_O (700 μl, 1:1, v/v) and loaded onto a 1 ml SepPak strong anion exchange cartridge (Supleco) pre-equilibrated with C_2_H_5_OH/H_2_O (1:1, v/v). The column was washed with 2 ml of C_2_H_5_OH, and the eluate collected, dried and partitioned between the two phases arising from a mixture of *n*-butanol (3 ml) and water (3 ml). The resulting organic phase was recovered after centrifugation at 3500 *g*, and the aqueous phase again extracted twice with 3 ml of water-saturated butanol. The pooled extracts were back-washed twice with *n*-butanol-saturated water (3 ml). The *n*-butanol fraction was dried and re-suspended in 200 μl of *n*-butanol. The extracted radiolabelled material was quantified by liquid scintillation counting using 10% of the labelled material and 5 ml of EcoScintA (National Diagnostics, Atlanta, GA). The incorporation of [^14^C]Man*p* was determined by subtracting counts present in control assays (incubations in the absence of acceptor), which were typically less than 100 c.p.m. per assay. The remaining labelled material was subjected to TLC using silica gel plates (5735 silca gel 60F254, Merck) developed in CHCl_3_:CH_3_OH:H_2_O:NH_4_OH (65:25:3.6:0.5, v/v/v/v) and the products visualized by phosphorimaging (Kodak K Screen).

### Selective cleavage by partial acetolysis

[^14^C]-mannosylated products were dried and acetylated using 40 μl of pyridine/acetic anhydride (1:1, v/v) for 30 min at 100°C. The products were dried in a Speed Vac and residual acetic acid removed by co-evaporation with toluene (2 × 50 μl). The per-O-acetylated products were dissolved in 30 μl of acetic anhydride/acetic acid/sulphuric acid (10:10:1, v/v/v) and acetolysis performed for 8 h at 37°C (Brown *et al*., 1997). The reaction mixture was then quenched by the addition of 10 μl pyridine and 500 μl H_2_O. After 1 h, the per-O-acetylated products were recovered by extraction into CHCl_3_. The CHCl_3_ phase was washed three times with 500 μl of H_2_O and dried. The products were then de-O-acetylated using 200 μl of concentrated ammonium hydroxide/methanol (1:1, v/v) for 60 h at 37°C and subsequently dried. The acetolysis products derived from α-D-[^14^C]Man*p*-(1→6)-α-D-Man*p*-(1→6)-α-D-Man*p-O*-C_8_ were re-dissolved in 40% propan-1-ol and analysed by TLC using one development with propan-1-ol/acetone/H_2_O (5:4:1, v/v/v), followed by one development with butan-1-ol/acetone/H_2_O (5:3.5:1.5, v/v/v) and the products visualized by phosphorimaging (Kodak K Screen). The acetolysis products derived from the CHCl_3_/CH_3_OH/H_2_O (10:10:3)-soluble [^14^C]-labelled mannooligosaccharide polymer [α(1→6)-linear-LM-A and α(1→6)-linear-LM-B] were re-dissolved in water and applied onto a 50 ml Bio-Gel P-2 gel filtration column (30 × 1.5 cm; Bio-Rad). Elution from the column was performed using water and 1 ml fractions collected which were subsequently quantified by liquid scintillation counting. The control de-O-acylated [^14^C]-labelled mannooligosaccharide polymers prior to acetolysis eluted form the Bio-Gel P-2 column at fractions 11–13 and degraded acetolysis products were retained and co-eluted in later fractions 33–39 based on a de-O-acylated PI[^14^C]M_2_, [^14^C]Man_1_GlcAGroAc_2_ and [^14^C]Man standards.

## Supplementary material

Figure S1 This material is available as part of the online article from: http://www.blackwell-synergy.com/doi/abs/10.1111/j.1365-2958.2008.06265.x (This link will take you to the article abstract). Please note: Blackwell Publishing is not responsible for the content or functionality of any supplementary materials supplied by the authors. Any queries (other than missing material) should be directed to the corresponding author for the article.

## References

[b1] Adams LB, Fukutomi Y, Krahenbuhl JL (1993). Regulation of murine macrophage effector functions by lipoarabinomannan from mycobacterial strains with different degrees of virulence. Infect Immun.

[b2] Alderwick LJ, Radmacher E, Seidel M, Gande R, Hitchen PG, Morris HR (2005). Deletion of Cg-*emb* in corynebacterianeae leads to a novel truncated cell wall arabinogalactan, whereas inactivation of Cg-*ubiA* results in an arabinan-deficient mutant with a cell wall galactan core. J Biol Chem.

[b3] Alderwick LJ, Seidel M, Sahm H, Besra GS, Eggeling L (2006a). Identification of a Novel arabinofuranosyltransferase (AftA) involved in cell wall arabinan biosynthesis in *Mycobacterium tuberculosis*. J Biol Chem.

[b4] Alderwick LJ, Dover LG, Seidel M, Gande R, Sahm H, Eggeling L, Besra GS (2006b). Arabinan-deficient mutants of *Corynebacterium glutamicum* and the consequent flux in decaprenylmonophosphoryl-D-arabinose metabolism. Glycobiology.

[b5] Appelmelk BJ, den Dunnen J, Driessen NN, Ummels R, Pak M, Nigou J (2007). The mannose cap of mycobacterial lipoarabinomannan does not dominate the *Mycobacterium*–host interaction. Cell Microbiol.

[b6] Bardarov S, Bardarov S, Pavelka MS, Sambandamurthy V, Larsen M, Tufariello J (2002). Specialized transduction: an efficient method for generating marked and unmarked targeted gene disruptions in *Mycobacterium tuberculosis*, *M. bovis* BCG and *M. smegmatis*. Microbiology.

[b7] Belanger AE, Besra GS, Ford ME, Mikusova K, Belisle JT, Brennan PJ, Inamine JM (1996). The embAB genes of *Mycobacterium avium* encode an arabinosyl transferase involved in cell wall arabinan biosynthesis that is the target for the antimycobacterial drug ethambutol. Proc Natl Acad Sci USA.

[b8] Berg S, Starbuck J, Torrelles JB, Vissa VD, Crick DC, Chatterjee D, Brennan PJ (2005). Roles of conserved proline and glycosyltransferase motifs of EmbC in biosynthesis of lipoarabinomannan. J Biol Chem.

[b9] Berg S, Kaur D, Jackson M, Brennan PJ (2007). The Glycosyltransferases of *Mycobacterium tuberculosis*; roles in the synthesis of arabinogalactan, lipoarabinomannan, and other glycoconjugates. Glycobiology.

[b10] Besra GS, Brennan PJ (1997). The mycobacterial cell wall: biosynthesis of arabinogalactan and lipoarabinomannan. Biochem Soc Trans.

[b11] Besra GS, Khoo KH, McNeil MR, Dell A, Morris HR, Brennan PJ (1995). A new interpretation of the structure of the mycolyl-arabinogalactan complex of *Mycobacterium tuberculosis* as revealed through characterization of oligoglycosylalditol fragments by fast-atom bombardment mass spectrometry and ^1^H nuclear magnetic resonance spectroscopy. Biochemistry.

[b12] Besra GS, Morehouse CB, Rittner CM, Waechter CJ, Brennan PJ (1997). Biosynthesis of mycobacterial lipoarabinomannan. J Biol Chem.

[b13] Bloom BR, Murray CJ (1992). Tuberculosis: commentary on a reemergent killer. Science.

[b14] Brennan PJ (2003). Structure, function, and biogenesis of the cell wall of *Mycobacterium tuberculosis*. Tuberculosis (Edinb).

[b15] Brennan P, Ballou CE (1967). Biosynthesis of mannophosphoinositides by *Mycobacterium phlei*. The family of dimannophosphoinositides. J Biol Chem.

[b16] Brennan P, Ballou CE (1968). Biosynthesis of mannophosphoinositides by *Mycobacterium phlei*. Enzymatic acylation of the dimannophosphoinositides. J Biol Chem.

[b17] Brennan PJ, Nikaido H (1995). The envelope of mycobacteria. Annu Rev Biochem.

[b18] Brown JR, Guther MLS, Field RA, Ferguson MAJ (1997). Hydrophobic mannosides act as acceptors for trypanosome α-mannosyltransferases. Glycobiology.

[b19] Brown JR, Field RA, Barker A, Guy M, Grewal R, Khoo KH (2001). Synthetic mannosides act as acceptors for mycobacterial α1–6 mannosyltransferase. Bioorg Med Chem.

[b20] Chatterjee D, Khoo KH (1998). Mycobacterial lipoarabinomannan: an extraordinary lipoheteroglycan with profound physiological effects. Glycobiology.

[b21] Chatterjee D, Khoo KH, McNeil MR, Dell A, Morris HR, Brennan PJ (1993). Structural definition of the non-reducing termini of mannose-capped LAM from *Mycobacterium tuberculosis* through selective enzymatic degradation and fast atom bombardment-mass spectrometry. Glycobiology.

[b22] Coyle MB, Lipsky BA (1990). Coryneform bacteria in infectious diseases: clinical and laboratory aspects. Clin Microbiol Rev.

[b23] Daffé M, Brennan PJ, McNeil M (1990). Predominant structural features of the cell wall arabinogalactan of *Mycobacterium tuberculosis* as revealed through characterization of oligoglycosyl alditol fragments by gas chromatography/mass spectrometry and by ^1^H and ^13^C NMR analyses. J Biol Chem.

[b24] Daffe M, McNeil M, Brennan PJ (1993). Major structural features of the cell wall arabinogalactans of *Mycobacterium*, *Rhodococcus*, and *Nocardia* spp. Carbohydr Res.

[b25] Dinadayala P, Kaur D, Berg S, Amin AG, Vissa VD, Chatterjee D (2006). Genetic basis for the synthesis of the immunomodulatory mannose caps of lipoarabinomannan in *Mycobacterium tuberculosis*. J Biol Chem.

[b26] Dobson G, Minnikin DE, Minnikin SM, Parlett JH, Goodfellow M, Goodfellow M, Minnikin DE (1985). Systematic analysis of complex mycobacterial lipids. Chemical Methods in Bacterial Systematics.

[b27] Dover LG, Cerdeno-Tarraga AM, Pallen MJ, Parkhill J, Besra GS (2004). Comparative cell wall core biosynthesis in the mycolated pathogens, *Mycobacterium tuberculosis* and *Corynebacterium diphtheriae*. FEMS Microbiol Rev.

[b28] Eggeling L, Bott M (2005). Handbook of Corynebacterium Glutamicum.

[b29] Eng BH, Guerinot ML, Eide D, Saier MH (1998). Sequence analyses and phylogenetic characterization of the ZIP family of metal ion transport proteins. J Membr Biol.

[b30] Fratti RA, Chua J, Vergne I, Deretic V (2003). *Mycobacterium tuberculosis* glycosylated phosphatidylinositol causes phagosome maturation arrest. Proc Natl Acad Sci.

[b31] Funke G, von Graevenitz A, Clarridge JE, Bernard KA (1997). Clinical microbiology of coryneform bacteria. Clin Microbiol Rev.

[b32] Gande R, Gibson KJ, Brown AK, Krumbach K, Dover LG, Sahm H (2004). Acyl-CoA carboxylases (accD2 and accD3), together with a unique polyketide synthase (Cg-pks), are key to mycolic acid biosynthesis in *Corynebacterianeae* such as *Corynebacterium glutamicum* and *Mycobacterium tuberculosis*. J Biol Chem.

[b33] Gibson KJ, Eggeling L, Maughan WN, Krumbach K, Gurcha SS, Nigou J (2003). Disruption of Cg-Ppm1, a polyprenyl monophosphomannose synthase, and the generation of lipoglycan-less mutants in *Corynebacterium glutamicum*. J Biol Chem.

[b34] Gilleron M, Himoudi N, Adam O, Constant P, Venisse A, Riviere M, Puzo G (1997). *Mycobacterium smegmatis* phosphoinositols-glyceroarabinomannans. Structure and localization of alkali-labile and alkali-stable phosphoinositides. J Biol Chem.

[b35] Guerardel Y, Maes E, Elass E, Leroy Y, Timmerman P, Besra GS (2002). Structural study of lipomannan and lipoarabinomannan from *Mycobacterium chelonae*. Presence of unusual components with α1,3-mannopyranose side chains. J Biol Chem.

[b36] Gurcha SS, Baulard AR, Kremer L, Locht C, Moody DB, Muhlecker W (2002). Ppm1, a novel polyprenol monophosphomannose synthase from *Mycobacterium tuberculosis*. Biochem J.

[b37] Hill DL, Ballou CE (1966). Biosynthesis of mannophospholipids by *Mycobacterium phlei*. J Biol Chem.

[b38] Huet G, Castaing JP, Fournier D, Daffe M, Saves I (2006). Protein splicing of SufB is crucial for the functionality of the *Mycobacterium tuberculosis* SUF machinery. J Bacteriol.

[b39] Hunter SW, Gaylord H, Brennan PJ (1986). Structure and antigenicity of the phosphorylated lipopolysaccharide antigens from the leprosy and tubercle bacilli. J Biol Chem.

[b40] Kaur D, Berg S, Dinadayala P, Gicquel B, Chatterjee D, McNeil MR (2006). Biosynthesis of mycobacterial lipoarabinomannan: role of a branching mannosyltransferase. Proc Natl Acad Sci USA.

[b41] Kaur D, McNeil MR, Khoo KH, Chatterjee D, Crick DC, Jackson M, Brennan PJ (2007). New insights into the biosynthesis of mycobacterial lipomannan arising from deletion of a conserved gene. J Biol Chem.

[b42] Khoo KH, Dell A, Morris HR, Brennan PJ, Chatterjee D (1995). Inositol phosphate capping of the nonreducing termini of lipoarabinomannan from rapidly growing strains of *Mycobacterium*. J Biol Chem.

[b43] Knutson KL, Hmama Z, Herrera-Velit P, Rochford R, Reiner NE (1998). Lipoarabinomannan of *Mycobacterium tuberculosis* promotes protein tyrosine dephosphorylation and inhibition of mitogen-activated protein kinase in human mononuclear phagocytes. Role of the Src homology 2 containing tyrosine phosphatase 1. J Biol Chem.

[b44] Kordulakova J, Gilleron M, Mikusova K, Puzo G, Brennan PJ, Gicquel B, Jackson M (2002). Definition of the first mannosylation step in phosphatidylinositol mannoside synthesis. PimA is essential for growth of mycobacteria. J Biol Chem.

[b45] Kordulakova J, Gilleron M, Puzo G, Brennan PJ, Gicquel B, Mikusova K, Jackson M (2003). Identification of the required acyltransferase step in the biosynthesis of the phosphatidylinositol mannosides of *Mycobacterium* species. J Biol Chem.

[b46] Kremer L, Dover LG, Morehouse C, Hitchin P, Everett M, Morris HR (2001). Galactan biosynthesis in *Mycobacterium tuberculosis*. Identification of a bifunctional UDP-galactofuranosyltransferase. J Biol Chem.

[b47] Kremer L, Gurcha SS, Bifani P, Hitchen PG, Baulard A, Morris HR (2002). Characterization of a putative α-mannosyltransferase involved in phosphatidylinositol trimannoside biosynthesis in *Mycobacterium tuberculosis*. Biochem J.

[b48] Lea-Smith DJ, Martin KL, Pyke JS, Tull D, McConville MJ, Coppel RL, Crellin PK (2008). Analysis of a new mannosyltransferase required for the synthesis of phosphotidylinositol mannosides and lipoarabinomannan reveals two lipomannan pools in *Corynebacterineae*. J Biol Chem.

[b49] Liu J, Mushegian A (2003). Three monophyletic superfamilies account for the majority of the known glycosyltransferases. Protein Sci.

[b50] Lu G, Westbrooks JM, Davidson AL, Chen J (2005). ATP hydrolysis is required to reset the ATP-binding cassette dimer into the resting-state conformation. Proc Natl Acad Sci USA.

[b51] Ludwiczak P, Gilleron M, Bordat Y, Martin C, Gicquel B, Puzo G (2002). *Mycobacterium tuberculosis phoP* mutant: lipoarabinomannan molecular structure. Microbiology.

[b52] McNeil M, Daffe M, Brennan PJ (1990). Evidence for the nature of the link between the arabinogalactan and peptidoglycan of mycobacterial cell walls. J Biol Chem.

[b53] McNeil M, Daffe M, Brennan PJ (1991). Location of the mycolyl ester substituents in the cell walls of mycobacteria. J Biol Chem.

[b54] Maeda N, Nigou J, Herrmann JL, Jackson M, Amara A, Lagrange PH (2003). The cell surface receptor DC-SIGN discriminates between *Mycobacterium* species through selective recognition of the mannose caps on lipoarabinomannan. J Biol Chem.

[b55] Minnikin DE, Kremer L, Dover LG, Besra GS (2002). The methyl-branched fortifications of *Mycobacterium tuberculosis*. Chem Biol.

[b56] Mishra AK, Alderwick LJ, Rittmann D, Tatituri RV, Nigou J, Gilleron M (2007). Identification of an α(1–6) mannopyranosyltransferase (MptA), involved in *Corynebacterium glutamicum* lipomanann biosynthesis, and identification of its orthologue in *Mycobacterium tuberculosis*. Mol Microbiol.

[b57] Mishra AK, Klein C, Gurcha SS, Alderwick LJ, Babu P, Hitchen PG (2008). Structural characterization and functional properties of a novel lipomannan variant isolated from a *Corynebacterium glutamicum pimB′* mutant: Antonie Van Leeuwenhoek.

[b58] Morgenstern B (2004). DIALIGN: multiple DNA and protein sequence alignment at BiBiServ. Nucleic Acids Res.

[b59] Morita YS, Patterson JH, Billman-Jacobe H, McConville MJ (2004). Biosynthesis of mycobacterial phosphatidylinositol mannosides. Biochem J.

[b60] Morita YS, Sena CB, Waller RF, Kurokawa K, Sernee MF, Nakatani F (2006). PimE Is a Polyprenol-phosphate-mannose-dependent mannosyltransferase that transfers the fifth mannose of phosphatidylinositol mannoside in mycobacteria. J Biol Chem.

[b61] Nigou J, Gilleron M, Cahuzac B, Bounery JD, Herold M, Thurnher M, Puzo G (1997). The phosphatidyl-myo-inositol anchor of the lipoarabinomannans from *Mycobacterium bovis* bacillus Calmette Guerin. Heterogeneity, structure, and role in the regulation of cytokine secretion. J Biol Chem.

[b62] Nigou J, Gilleron M, Brando T, Vercellone A, Puzo G (1999). Structural definition of arabinomannans from *Mycobacterium bovis* BCG. Glycoconj J.

[b63] Nigou J, Gilleron M, Rojas M, Garcia LF, Thurnher M, Puzo G (2002). Mycobacterial lipoarabinomannans: modulators of dendritic cell function and the apoptotic response. Microbes Infect.

[b64] Nigou J, Gilleron M, Puzo G (2003). Lipoarabinomannans: from structure to biosynthesis. Biochimie.

[b65] Puech V, Guilhot C, Perez E, Tropis M, Armitige LY, Gicquel B, Daffe M (2002). Evidence for a partial redundancy of the fibronectin-binding proteins for the transfer of mycoloyl residues onto the cell wall arabinogalactan termini of *Mycobacterium tuberculosis*. Mol Microbiol.

[b66] Reizer J, Reizer A, Saier MH (1992). A new subfamily of bacterial ABC-type transport systems catalyzing export of drugs and carbohydrates. Protein Sci.

[b67] Schaeffer ML, Khoo KH, Besra GS, Chatterjee D, Brennan PJ, Belisle JT, Inamine JM (1999). The *pimB* gene of *Mycobacterium tuberculosis* encodes a mannosyltransferase involved in lipoarabinomannan biosynthesis. J Biol Chem.

[b68] Schafer A, Tauch A, Jager W, Kalinowski J, Thierbach G, Puhler A (1994). Small mobilizable multi-purpose cloning vectors derived from the *Escherichia coli* plasmids pK18 and pK19: selection of defined deletions in the chromosome of *Corynebacterium glutamicum*. Gene.

[b69] Schlesinger LS, Hull SR, Kaufman TM (1994). Binding of the terminal mannosyl units of lipoarabinomannan from a virulent strain of *Mycobacterium tuberculosis* to human macrophages. J Immunol.

[b70] Seidel M, Alderwick LJ, Birch HL, Sahm H, Eggeling L, Besra GS (2007). Identification of a novel arabinofuranosyltransferase AftB involved in a terminal step of cell wall arabinan biosynthesis in *Corynebacterianeae*, such as *Corynebacterium glutamicum* and *Mycobacterium tuberculosis*. J Biol Chem.

[b71] Soding J, Biegert A, Lupas AN (2005). The HHpred interactive server for protein homology detection and structure prediction. Nucleic Acids Res.

[b72] Stackebrandt E, Rainey FA, Ward-Rainey NL (1997). Proposal for a new hierarchic classification system, *Actinobacteria* classis nov. Int J Syst Bacteriol.

[b73] Tascon RE, Soares CS, Ragno S, Stavropoulos E, Hirst EM, Colston MJ (2000). *Mycobacterium tuberculosis*-activated dendritic cells induce protective immunity in mice. Immunology.

[b74] Tatituri RV, Illarionov PA, Dover LG, Nigou J, Gilleron M, Hitchen P (2007). Inactivation of *Corynebacterium glutamicum* NCgl0452 and the role of MgtA in the biosynthesis of a novel mannosylated glycolipid involved in lipomannan biosynthesis. J Biol Chem.

[b75] Vrljic M, Garg J, Bellmann A, Wachi S, Freudl R, Malecki MJ (1999). The LysE superfamily: topology of the lysine exporter LysE of *Corynebacterium glutamicum*, a paradyme for a novel superfamily of transmembrane solute translocators. J Mol Microbiol Biotechnol.

[b76] Wang C, Hayes B, Vestling MM, Takayama K (2006). Transposome mutagenesis of an integral membrane transporter in *Corynebacterium matruchotii*. Biochem Biophys Res Commun.

[b77] Zhang N, Torrelles JB, McNeil MR, Escuyer VE, Khoo KH, Brennan PJ, Chatterjee D (2003). The Emb proteins of mycobacteria direct arabinosylation of lipoarabinomannan and arabinogalactan *via* an N-terminal recognition region and a C-terminal synthetic region. Mol Microbiol.

