# Supplementary material for: Identification of a novel α(1→6) mannopyranosyltransferase MptB from Corynebacterium glutamicum by deletion of a conserved gene, NCgl1505, affords a lipomannan- and lipoarabinomannan-deficient mutant
Source: Mol Microbiol. 2008 Jun;68(6):1595–613. doi: 10.1111/j.1365-2958.2008.06265.x (PMC2440535; doi:10.1111/j.1365-2958.2008.06265.x)
Supplement: Figure S1 [file mmi0068-1595-SD1.pdf]

Supplementary Material

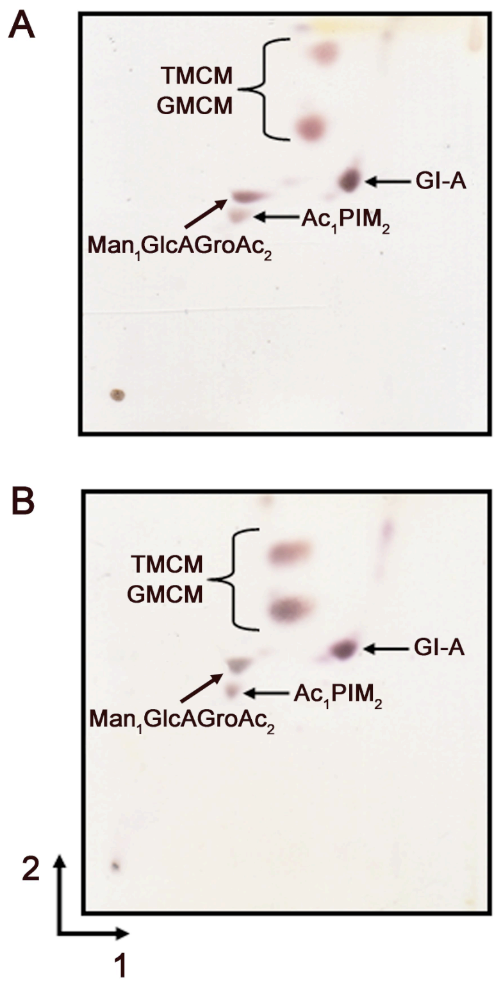

Supplementary Figure 1: Analysis of PIM biosynthesis in *C. glutamicum* and *C. glutamicum*Δ*mptB*. The polar lipid profiles of *C. glutamicum* **(A)** and *C. glutamicum*Δ*mptB* **(B)** are shown. The polar lipid extract was examined by 2D-TLC on aluminum-backed plates of silica gel 60 F_{254} (Merck 5554), using $CHCl_3/CH_3OH/H_2O$ (60:30:6, v/v/v) in the first direction and $CHCl_3/CH_3COOH/CH_3OH/H_2O$ (40:25:3:6, v/v/v/v) in the second direction. Glycolipids were visualized by spraying plates with α-naphthol/sulfuric acid, followed by gentle charring of the plates. Abbreviations: Gl-A, α-**D**-glucopyranosyluronic acid-(1→3)-glycerol; GMCM, glucose monocorynomycolate; TMCM, trehalose monocorynomycolate.
